# Monoclonal antibodies in type 2 asthma: a systematic review and network meta-analysis

**DOI:** 10.1186/s12931-019-1138-3

**Published:** 2019-08-08

**Authors:** Ahmed Edris, Silke De Feyter, Tania Maes, Guy Joos, Lies Lahousse

**Affiliations:** 1 Department of Bioanalysis, Pharmaceutical Care Unit, Ghent University, Ottergemsesteenweg 460, 9000 Ghent, Belgium; 2 Department of Internal Medicine and Pediatrics, Respiratory Medicine, Ghent University Hospital, Ghent, Belgium

**Keywords:** Asthma, Monoclonal antibodies, Exacerbations, Network meta-analysis

## Abstract

Since novel treatments to target eosinophilic inflammation in Type 2 asthma are emerging, we aimed to evaluate and meta-analyze the efficacy of monoclonal antibodies to reduce exacerbation rate. PubMed and Web of Science were searched for phase II and phase III randomized clinical trials with monoclonal antibodies targeting key mediators of type 2-associated asthma. Thirty trials were selected involving biologics that target the IL-5 pathway, IL-13, the common IL-4 and IL-13 receptor, IL-9, IL-2 and TSLP. As no head-to-head trials were retrieved from literature, we performed an arm-based network meta-analysis to compare effects on exacerbation rate between the different treatments.

Mepolizumab, reslizumab and benralizumab significantly reduced the risk of exacerbations compared to placebo (by 47–52%, 50–60%, and 28–51% respectively). Reslizumab and benralizumab also improved lung function. Dupilumab and tezepelumab improved lung function in frequent exacerbators. Lebrikizumab had no significant effect on the number of exacerbations, symptom control or health-related quality of life. Tralokinumab improved lung function compared to placebo. Network meta-analysis of all treatment and placebo arms, showed no superiority of one biologic over the others. Large reductions in exacerbation rates were observed compared to placebo, though only benralizumab was sufficiently powered (*n* = 2051) to demonstrate significantly decreased exacerbation rates in the subgroup analysis of IL-5 acting agents compared to placebo.

Monoclonal antibodies such as mepolizumab, reslizumab and benralizumab have proven their benefit to reduce exacerbation rates in severe persistent eosinophilic asthma in the published trials. However, no statistically significant superiority was observed of one biologic over the other in the network meta-analysis. More studies with direct head to head comparisons and better defined endotypes are required.

**Electronic supplementary material:**

The online version of this article (10.1186/s12931-019-1138-3) contains supplementary material, which is available to authorized users.

## Introduction

The Global Initiative of Asthma (GINA), defines asthma as a disorder of lower airways, usually associated with chronic airway inflammation characterized by episodic respiratory symptoms such as wheeze, shortness of breath, chest tightness and cough, together with variable expiratory airflow obstruction [1]. Asthma is an overarching term for a heterogeneous disease including multiple underlying disease mechanisms with common clinical symptoms [2]. The estimated global prevalence of clinical asthma in adults is 4.5%, translating into over 300 million people worldwide with asthma. The prevalence is higher in developed countries, up to 21.5% [3].

The severity of the asthma symptoms and airflow limitation typically varies over time. Symptoms often worsen at night or early in the morning. Fluctuations can be caused by specific triggers such as allergens, as well as non-specific triggers such as exercise, laughter, irritant exposure, cold air and viral infections. Conversely, asthma symptoms can disappear spontaneously for weeks or months. Life-threatening exacerbations, defined as acute episodic flare-ups are the most important complications of the disease, affecting morbidity and mortality [1].

Asthma-phenotypes can be categorized based on clinical characteristics or environmental triggers. This approach evolved towards defining subtypes by different underlying biology, also called ‘endotypes’ [2, 4]. Based on this pathobiology-stratified approach, asthma patients can be subdivided into two main categories: patients with type 2 asthma and patients with non-type 2 asthma [5]. Eosinophilic inflammation is the hallmark symptom of type 2 asthma [2]. Blood or sputum eosinophilia are associated with a higher risk of severe asthma exacerbations [6]. The most important differentiating factors between the different subendotypes are age at disease onset and atopy [7].

Treatment goals in asthma include symptom control and reducing risk of future exacerbations. However, approximately 3 to 5% of asthmatic patients have severe asthma where either symptoms persist or numerous exacerbations occur despite maximal treatment, an estimate that varies by country and may reach ≥10% in the United States [8, 9]. Therefore, an alternative approach is required, guided by the underlying inflammatory pathway or endotype [10]. According to the GINA pocket guide for the management of difficult-to-treat and severe asthma, type 2 inflammation should be considered if any of the following are found in a patient taking high-dose inhaled corticosteroids (ICS): elevated blood eosinophils (≥150/μL), elevated sputum eosinophils (≥2%), elevated FeNO (≥20 ppb) or asthma that is clinically allergen-driven [10].

T_H_2-high asthmatics have an overall favorable therapy response to ICS [11]. Nevertheless, a notable subgroup of those patients may require higher doses, oral corticosteroids or have persistent symptoms despite regular corticosteroid use [12]. Therefore, several monoclonal antibodies targeting specific inflammatory pathways have been developed to tackle this issue [11]. Blocking TSLP, CCR3, IL-5, PGD2, IL-4, IL-13, IL-9 and/or IgE may be effective in the treatment of allergic eosinophilic asthma [13–21].

This systematic review aimed to investigate the endotype-guided asthma treatment possibilities by monoclonal antibodies, focusing on the key drivers of eosinophilic inflammation in type 2-associated adult asthmatics. We aimed to provide a clear overview of the currently available or emerging monoclonal antibodies in asthma. We subsequently compared the results from different trials to evaluate the effects of monoclonal antibodies on the median exacerbation rate.

## Methods

We conducted this review according to a predefined protocol compliant with the PRISMA guidelines for systematic reviews [22]. The protocol registration was performed using the PROSPERO international prospective register of systematic reviews. (Registration number: CRD42019127706) A structured search strategy of PubMed and Web of Science was developed to identify all phase II and III clinical trials published in English, investigating the treatment of type 2 asthma using monoclonal antibodies between 2005 and 2018. Studies conducted on small sample sizes (50 or less), post-hoc studies, open label extensions and studies taking glucocorticoid sparing effect as primary endpoint were excluded [23–26]. Conference or poster abstracts and studies not conducted on humans were also excluded. These exclusions aimed to ensure adequate power, homogeneity and clinical relevance among included studies. Studies conducted on omalizumab, an immunoglobulin E neutralizing agent, were excluded, as it is currently a well-known and established therapeutic target, and an excellent Cochrane systematic review discussing its use in adult and children has already been published [27]. Search results were reviewed independently by two investigators (AE and SDF) to determine the eligibility of potential studies, results were compared and disagreement was resolved to create the final list of included studies by the involvement of a third researcher (LL). Risk of bias was assessed using the Cochrane risk of bias tool for randomized controlled trials [28]. This tool assesses studies based on six criteria including: random sequence generation, allocation concealment, selective reporting, blinding, incomplete outcome data and a category for any other perceived type of bias [28]. The search terms used in both databases and details on data extraction strategy are included in the Additional file 1: online supplement.

### Statistical analysis

Arm-based patient centered network meta-analysis was conducted using the pcnetmeta package in R statistical software. With no head to head trials available, arm-based network meta-analysis estimates the comparative effect for multiple interventions based on their pooled effects from the included studies [29–32]. Specific details regarding the analysis methods are outlined in the Additional file 1: online supplement.

## Results

**Fig. 1:**
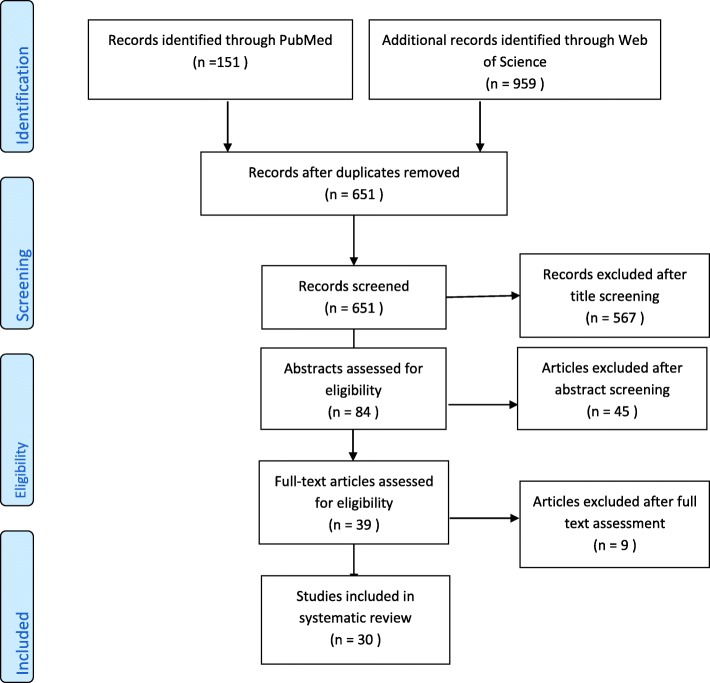
Search through PubMed and Web of Science: flow chart. Flowchart of the search and selection procedure. Of 651 initial records (after duplicate removal) initially retrieved with our search strategy, 567 studies met our exclusion criteria based on title, and 45 based on abstracts. A full text review was performed for the remaining 39 studies, and nine were excluded

We initially identified 1110 records, after removal of duplicates, 651 articles remained. 84 articles were eligible for abstract review based on title, subsequently 39 articles were selected for full text review based on abstract. Thirty records were included in the systematic review. A flow diagram of the selection procedure is represented in Fig. 1. Results focus on exacerbations for which 13 records were included in the meta-analysis [13, 15, 19, 33–42]. Additional results on lung function, quality of life and safety are outlined in the Additional file 1: online supplement.

### Risk of Bias

The included studies had a limited risk of bias (Table 1). Fifteen (50%) of the included studies had a low risk of bias, 9 studies (30%) had a moderate risk of bias and 6 studies (20%) had a high risk of bias. High or moderate risk of bias was mainly due to unreported randomization (*n* = 6), allocation concealment (*n* = 13) or blinding techniques (*n* = 7). All trials except one were industry funded, this was regarded as an unclear potential of bias, considered higher if company staff were involved in trial design or data analysis [43–45]. One trial was deemed to have a high risk of bias in their reporting [46].

**Table 1:** Risk of Bias for all studies included in the systematic review

Study (Ref)	Random Sequence Generation	Allocation Concealment	Selective Reporting	Other Bias	Blinding	Incomplete outcome data	BIAS judgement
*Flood-Page* et al. *2007* [50]	Unclear	Unclear	Low	High	Unclear	Low	High Risk
*Busse* et al. *2008* [68]	Unclear	Unclear	Low	Unclear	Low	Low	High Risk
*Haldar* et al. *2009* [49]	Low	Unclear	Low	Unclear	Unclear	Low	Intermediate Risk
*Corren* et al. *2011* [18]	Low	Low	Low	Unclear	Low	Low	Low Risk
*Castro* et al. *2011* [54]	Unclear	Unclear	Low	Unclear	Low	Low	Intermediate Risk
*Pavord* et al. *2012* [15]	Low	Low	Low	Unclear	Low	Low	Low Risk
*Noonan* et al. *2013* [57]	Unclear	Unclear	Low	Unclear	Unclear	Low	High Risk
*Wenzel* et al. *2013* [65]	Low	Low	Low	Unclear	Low	Low	Low Risk
*Oh* et al. *2013* [20]	Low	Low	Unclear	Unclear	Low	Unclear	Low Risk
*Piper* et al. *2013* [59]	Low	Unclear	Low	Unclear	Unclear	Low	Intermediate Risk
*De Boever* et al. *2014* [62]	Low	Low	Low	Unclear	Low	Low	Low Risk
*Ortega* et al. *2014* [36]	Low	Unclear	Low	Unclear	Low	Unclear	Intermediate Risk
*Castro* et al. *2014* [42]	Low	Low	Low	Unclear	Low	Low	Low Risk
*Hanania* et al. *2015* [33]	Low	Low	Unclear	Unclear	Low	Low	Intermediate Risk
*Brightling* et al. *2015* [61]	Low	Low	Low	Unclear	Unclear	Low	Intermediate Risk
*Castro* et al. *2015* [37]	Low	Low	Low	Unclear	Low	Low	Low Risk
*Hanania* et al. *2016* [34]	Low	Low	Low	Unclear	Low	Low	Low Risk
*Bjermer* et al. *2016* [53]	Unclear	Unclear	Unclear	Unclear	Unclear	Unclear	High Risk
*Corren* et al. *2016* [52]	Unclear	Unclear	Low	Unclear	Unclear	Low	High Risk
*Bleecker* et al. *2016* [38]	Low	Unclear	Low	Unclear	Low	Low	Intermediate Risk
*FitzGerald* et al. *2016* [39]	Low	Low	Low	Unclear	Low	Low	Low Risk
*Park* et al. *2016* [40]	Low	Unclear	Low	Low	Low	Low	Low Risk
*Nowak* et al. *2016* [41]	Low	Unclear	Low	Unclear	Low	Low	Intermediate Risk
*Wenzel* et al. *2016* [64]	Low	Low	Low	Unclear	Low	Low	Low Risk
*Corren* et al. *2017* [13]	Low	Low	Low	Unclear	Low	Low	Low Risk
*Chupp* et al. *2017* [48]	Low	Low	Low	Unclear	Low	Low	Low Risk
*Ferguson* et al. *2017* [46]	Low	Low	Low	Unclear	Low	High	Intermediate Risk
*Panettieri* et al. *2018* [35]	Low	Low	Low	Unclear	Low	Low	Low Risk
*Russel* et al. *2018* [60]	Low	Low	Low	Unclear	Low	Low	Low Risk
*Castro* et al. *2018* [19]	Low	Unclear	Low	High	Low	Low	High Risk

Risk of bias assessed by Cochrane tool for randomized controlled trials. Thirty studies were assessed for their risk of bias. Underlined studies are included in the meta-analysis

### Targeting interleukin-5

**Fig. 2:**
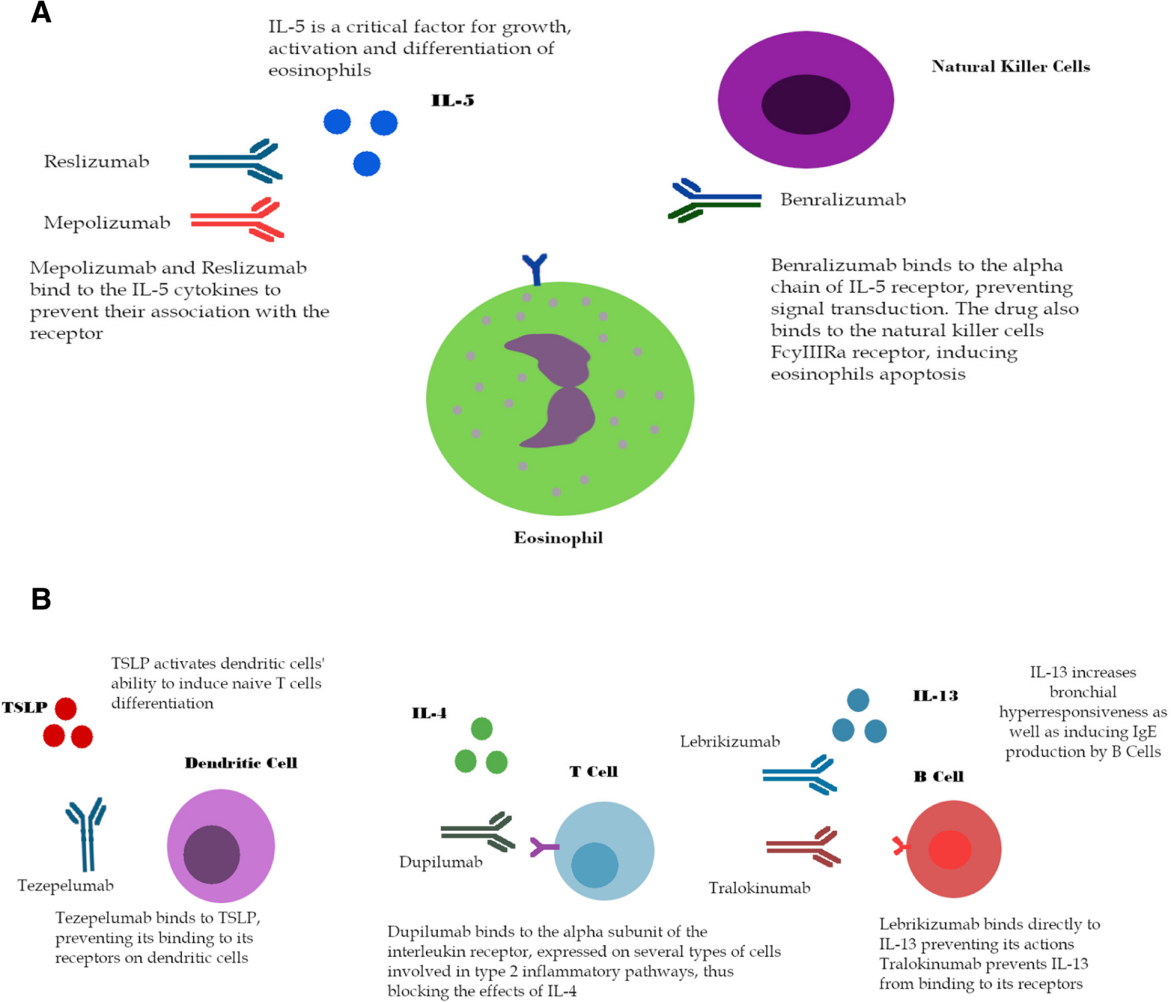
Mechanism of action of agents included in the meta-analysis. Mechanisms of action of monoclonal antibodies included in our meta-analysis (**a**) IL-5 is a critical factor for growth, differentiation and activation of eosinophils. Mepolizumab and reslizumab act as antibodies to IL-5 cytokines, binding to them and preventing their association with the receptor. Benralizumab is an IL-5 receptor blocker. It binds to the alpha chain of the IL-5 receptor (IL-5Rα), expressed on eosinophils. The antibody’s Fc domain binds to the FcγRIIIa domain, expressed on natural killer cells, which induces eosinophils’ apoptosis. **b** TSLP is an important cytokine in the inflammatory cascade, as it activates dendritic cells, inducing inflammatory reactions through their effects on T cells differentiation. Tezepelumab inhibits TSLP effects by binding to the cytokine. IL-4 is a potent inducer for T_H_2 cells differentiation, existing on several types of immune cells. Dupilumab binds to the alpha subunit of IL-4 receptor, inhibiting its effects. Lebrikizumab binds to IL-13 cytokines, and tralokinumab binds to its receptor on B cells, inhibiting its effects on IgE production

IL-5 is a key factor in the maturation and maintenance of eosinophils, potentially representing an interesting treatment target. Risk of exacerbations may be reduced by eosinophil elimination in inflammatory tissues and blood (Additional file 1: Table S1) [6, 47]. Several monoclonal antibodies acting on the pathway have been investigated and three agents have already received FDA and EMA approval for use in eosinophilic asthma (mepolizumab, benralizumab and reslizumab). The mechanisms of action of IL-5 agents are illustrated in Fig. 2.

#### Mepolizumab

Mepolizumab binds to soluble IL-5 inhibiting its interaction with its eosinophil surface receptor. It can be administered intravenously (IV) or subcutaneously (SC) [15, 36, 48–50]. Mepolizumab efficacy has been investigated in 5 trials. Patients who had at least 2 exacerbations in the previous year despite receiving high-dosage ICS were included in all trials, four trials required subjects to also have elevated blood eosinophil counts of ≥300 cells/μL or sputum eosinophil counts ≥3% [15, 36, 48–50]. IL-5, eosinophilia and exacerbations are closely linked, therefore, the effect of mepolizumab on yearly exacerbation rates has been extensively studied. Exacerbation risk was reduced compared to placebo by 53% for SC mepolizumab and 47 to 48% for low dose, 39% for medium dose and 49 to 52% for high dose IV mepolizumab [15, 36, 49]. Chupp et al. also reported a statistically significant reduction in clinically significant exacerbations (RR: 0.32, CI: 0.31–0.56) [48]. Flood-Page et al. report a non-statistically significant trend towards decreased exacerbation rate with a high dose of 750 mg IV, but their study did not select patients based on number of exacerbations [50]. Results about the reduction in severe exacerbations (defined as requiring hospitalization or emergency department visit) were less consistent [15, 36, 48, 49]. For example, Chupp et al. reported a significant reduction in exacerbations requiring admission to a hospital or ER visit, while the reduction in exacerbations requiring admissions as a single end-point was not significant [48]. Conversely, Ortega et al. reported reductions in both outcomes did not reach statistical significance [36].

#### Reslizumab

Reslizumab is administered IV, it binds to IL-5 selectively downregulating its pathway [51]. Reslizumab’s efficacy was investigated by four trials on different patient populations. Castro et al., in a phase III trial, only included patients with uncontrolled asthma who had at least 1 exacerbation in the previous year despite treatment with medium or high dose ICS and with blood eosinophil counts ≥400 cells/μL [37]. Two phase III trials did not take exacerbation history into account and differed in their inclusion of patients with lower blood eosinophil counts [52, 53]. An older phase II trial by Castro et al. had high dose ICS and sputum eosinophil counts ≥3% as main inclusion criteria [54]. Therefore, it is difficult to compare the four trial results. In our review, we included patients who received IV reslizumab 3.0 mg/kg every 4 weeks, as the 0.3 mg/kg proved less efficient [37, 52–54]. Castro et al., confirmed the beneficial effect of eosinophil reduction on exacerbation rates, exacerbation risk was reduced by 50–60% compared to placebo in the selected asthma population [37]. Reslizumab also delayed the time to the first exacerbation. A statistically non-significant decrease in hospital admissions or emergency department visits by asthma exacerbations was reported [37].

#### Benralizumab

In contrast to reslizumab and mepolizumab, benralizumab binds the interleukin-5 receptor α (IL-5Rα) expressed on eosinophil surfaces, as well as FcγIIIRa receptors located on natural killer cells leading to eosinophil apoptosis [55]. Benralizumab is usually administered SC. Efficacy has been investigated by two phase II [40, 41] and four phase III trials [38, 39, 42, 46]. Phase III trials selected patients with uncontrolled asthma who had at least 2 exacerbations in the previous year despite receiving high dose ICS + LABA and with blood eosinophil counts ≥300 cells/μL [38, 39, 42, 46].

The reduced eosinophil counts by benralizumab treatment improved exacerbation rates. According to the SIROCCO and the CALIMA-trial, risk of exacerbations was 36 to 45% lower compared to placebo when 30 mg benralizumab was administered every 4 weeks (Q4W) and 28 to 51% lower when benralizumab is administered every 8 weeks (Q8W). Patients who had 3 exacerbations or more in the previous year had most benefit from benralizumab treatment [38, 39]. Park et al. demonstrated a reduction in exacerbation rates compared to placebo of 33, 36 and 45% by 2 mg, 20 mg and 100 mg of benralizumab treatment every 4 weeks (every 8 weeks after first three doses), respectively [40]. Castro et al. also showed reduced exacerbation rates in the 100 mg dose groups vs placebo (0·34 vs 0·57) using the same dosing intervals [42]. Moreover, benralizumab reduced the risk of onset of exacerbation by 37 to 39% in the Q4W-arm and by 27 to 40% in the Q8W-arm [38, 40]. Inconsistent data was reported about benralizumab reducing exacerbation-related emergency department visits or hospital admissions compared to placebo [38, 39]. One investigation was designed to discover the potential to reduce future exacerbations by giving benralizumab IV during an acute exacerbation at the emergency department. The number of patients who experienced a second exacerbation within 12 weeks after the first was not reduced. However, the exacerbation rates and the number of exacerbation-related hospitalizations were 49 and 60% lower, respectively [41].

### Targeting interleukin-13

IL-13 may be the driver of goblet cell hyperplasia and smooth muscle contractility in type 2-associated asthma. Moreover, IL-13 is one of the two crucial cytokines in the isotype switch of B cells towards IgE in allergic asthma. Therefore, IL-13 may be a potential therapeutic target in the treatment of asthma (Additional file 1: Table S2) [47, 56].

#### Lebrikizumab

Lebrikizumab, administered SC, potentially improves lung function and symptom control in asthma by binding to IL-13, neutralizing its functional activities [18, 33, 34, 57]. Lebrikizumab’s efficacy has been investigated by three phase II and one phase III trials. Patients were included based on their maintenance ICS: low to high dose [18], medium to high dose (LUTE and VERSE-trials) [33], or no glucocorticoids at all [57]. The combined LUTE and VERSE-trials were originally set up to be phase III, however, an identified host cell protein impurity led to early termination of dosing and the protocol was amended as a phase II trial [33]. LAVOLTA I and LAVOLTA II were the only phase III trials to investigate lebrikizumab, eligible patients were aged 18–75 years with uncontrolled asthma, pre-bronchodilator FEV1 40–80% predicted, bronchodilator response of at least 12%, and on stable background therapy with inhaled corticosteroids for at least 6 months and at least one additional controller medication [34].

Lebrikizumab effect on exacerbation rate was inconsistent amongst the trials, The LUTE and VERSE replicate trials show significant reduced risk on exacerbations compared to placebo in the periostin-high group of patients treated with 37.5 mg (81% reduction) or 125 mg lebrikizumab (77% reduction). No significant reduced exacerbation rates are observed in periostin-high patients treated with lebrikizumab 250 mg and serum periostin-low patients irrespective of dose [33]. No consistent significant effect of lebrikizumab on exacerbation rates was reported in LAVOLTA I and LAVOLTA II phase III [34].

#### Tralokinumab

Tralokinumab inhibits downstream IL-13 mediated effects by preventing IL-13 binding to both IL-13Rα1 and IL-13Rα2, considered important mediators of fibrosis [58]. It is administered SC. Its efficacy was investigated in 3 phase II trials selecting patients with uncontrolled asthma who had at least one [59], three [60] or 2 to six exacerbations in the previous year [61]. Tralokinumab was also tested in STRATOS I and STRATOS II phase III trials that enrolled patients 12–75 years with a history of asthma for at least one year and requiring medium to high dose ICS and a LABA for at least 3 months before enrollment [35].

The primary outcome of the first phase II trial was the change in ACQ-6 after 13 weeks tralokinumab treatment. None of the administered doses improved symptom control compared to placebo [59]. Furthermore, none of the secondary outcomes (FEV_1_, FVC, PEF, exacerbations and AQLQ) significantly improved compared to placebo. The only exception was the improved prebronchodilator FEV_1_ compared to placebo by 600 mg tralokinumab treatment (0.20 L) that just reached significance [59]. Tralokinumab lack of clinical efficacy is further confirmed by the 2015 phase IIb randomized trial [61] as well as the recent 2018 STRATOS I and STRATOS II phase III trials, which showed inconsistent effects on exacerbation rate [35]. MESOS trial also showed no effect on bronchial eosinophilic count [60].

#### GSK679586

GSK679586 also binds and neutralizes IL-13 and is administered IV. The efficacy of GSK679586 is investigated by a phase II trial selecting patients with uncontrolled asthma despite receiving a high dose ICS [62].

The primary outcome was change in ACQ-7 after 12 weeks of therapy. No significant improvements in symptom control were demonstrated, even with increased serum IgE levels or elevated blood eosinophil counts [62]. GSK679586 did not result in statistically significant improvements in FEV_1_ [62]. Likewise, GSK679586 had no effects on exacerbations compared to placebo [62]. IgE levels in the intervention group remained generally unchanged during the treatment. Blood eosinophil counts where slightly higher compared to placebo because of a downward trend in eosinophil counts in the placebo group [62].

### Targeting both interleukin-4 and interleukin-13

IL-4 and IL-13 share a common receptor, IL-4Rα, an interesting therapeutic target: both powerful mediators of type 2 immunity are targeted by only one intervention. T-cell differentiation to the T_H_2-subtype, the isotype switch towards IgE and effects on goblet cell hyperplasia and smooth muscle contractility are prevented by blocking IL-4 and IL-13 simultaneously, which may result in improved asthma outcomes [47, 56, 63].

#### Dupilumab

Dupilumab is an anti-IL-4Rα antibody approved which binds to IL-4 type 1 receptor and is SC administered [19, 64, 65]. Dupilumab was recently approved by the FDA as an add-on maintenance therapy in moderate to severe asthma [66]. The efficacy of dupilumab treatment is mostly substantiated by a large-scale phase III trial (LIBERTY ASTHMA QUEST) for patients with uncontrolled asthma who had at least 1 exacerbation in the previous year despite treatment with high dose of ICS (Additional file 1: Table S3) [19]. Two older phase II trials primarily analyzed patients receiving medium-to high dose ICS + LABA and with blood eosinophil counts ≥300 cells/μL or sputum eosinophil counts ≥3% [64, 65].

The number of exacerbations is significantly reduced by 46.9 to 70.5% when dupilumab 200 mg or 300 mg is administered every two weeks, irrespective of eosinophil levels. The eosinophil-high patients and the FeNO-high patients showed better responses [19, 64]. Patients with blood eosinophil levels < 150/μL had exacerbation rates similar to those treated with placebo [19]. Weekly dupilumab administration resulted in an 87% reduction of asthma events [65]. The administration interval of 4 weeks turns out to be less advantageous, with small or non-significant reductions in annualized exacerbation rates [64]. In the overall population, dupilumab given every 2 weeks reduced exacerbation-related hospitalization or emergency department visit with 46.8% [19] and delayed the time to first exacerbation [64].

### Targeting interleukin-9

IL-9 is believed to have a mediating role in the pathogenesis of allergic asthma, especially in the mast cell component. Therefore, targeting IL-9 may be interesting in the hunt for newer and more specific asthma treatment strategies [47, 56].

#### MEDI-528

MEDI-528 targets IL-9 aiming to inhibit its function in the asthma pathogenesis. MEDI-528 is administered SC. A phase II trial investigated efficacy in patients with uncontrolled asthma who had at least 1 exacerbation in the previous year (Additional file 1: Table S4) [20].

The primary outcome, ACQ-6 at week 13 was not significantly affected by MEDI-528 treatment. Post-hoc analyses in subgroups stratifying patients based on atopy, ICS dose or peripheral blood eosinophil counts, showed no significant outcome. Likewise, no secondary outcomes were significantly improved: prebronchodilator FEV_1_, annualized exacerbation rate and AQLQ(S)-score [20].

### Targeting thymic stromal lymphopoietin

TSLP is one of the key drivers of the asthmatic pathophysiology as it is produced by the airway epithelium in response to inhaled allergens and proinflammatory stressors. Targeting TSLP may be interesting because of its upstream role in the asthma cascade [47, 67].

#### Tezepelumab

Tezepelumab binds to TSLP, inhibiting its stimulating activity on dendritic cells and innate lymphoid cells thus preventing the induction of type 2 cytokines (e.g.: IL-5, IL-4 and IL-13). It is administered SC. It has been investigated by a phase II trial in patients with uncontrolled asthma and multiple exacerbations in the previous year despite receiving medium to high dose ICS (Additional file 1: Table S5) [13]. The exacerbation risk was significantly reduced in tezepelumab groups - irrespective of the baseline blood eosinophil count - compared to placebo by 62% in the low-dose group, 71% in the medium-dose group and 66% in the high-dose group.

#### Daclizumab

Daclizumab works by binding to the IL-2R α chain (CD25) thereby inhibiting lymphocyte activation. Only one RCT was retrieved from the literature for Daclizumab (Additional file 1: Table S6). Busse et al., in 2008, tested the efficacy of Daclizumab in 115 patients assessed by the change in FEV1 in moderate to severe uncontrolled asthma [68]. Improvements were noted for the intervention group (88 patients) (4.4 ± 1.80% vs 1.5 ± 2.39%; *p* = 0.05), daytime asthma symptoms were reduced (*p* = 0.018), and time to exacerbation was prolonged (*p* = 0.024). FEV1 absolute increase (L) in the treated group ranged from 2.34 ± 0.07 (baseline) to 2.4 ± 0.08 (Day 84), while patients receiving placebo had a decrease in FEV1 from 2.25 ± 0.1 to 2.2 ± 0.1 L. [68]. The trial reported an increase in serious adverse events in the treatment arm (5 vs 1) [68].

#### Network meta-analysis

**Fig. 3:**
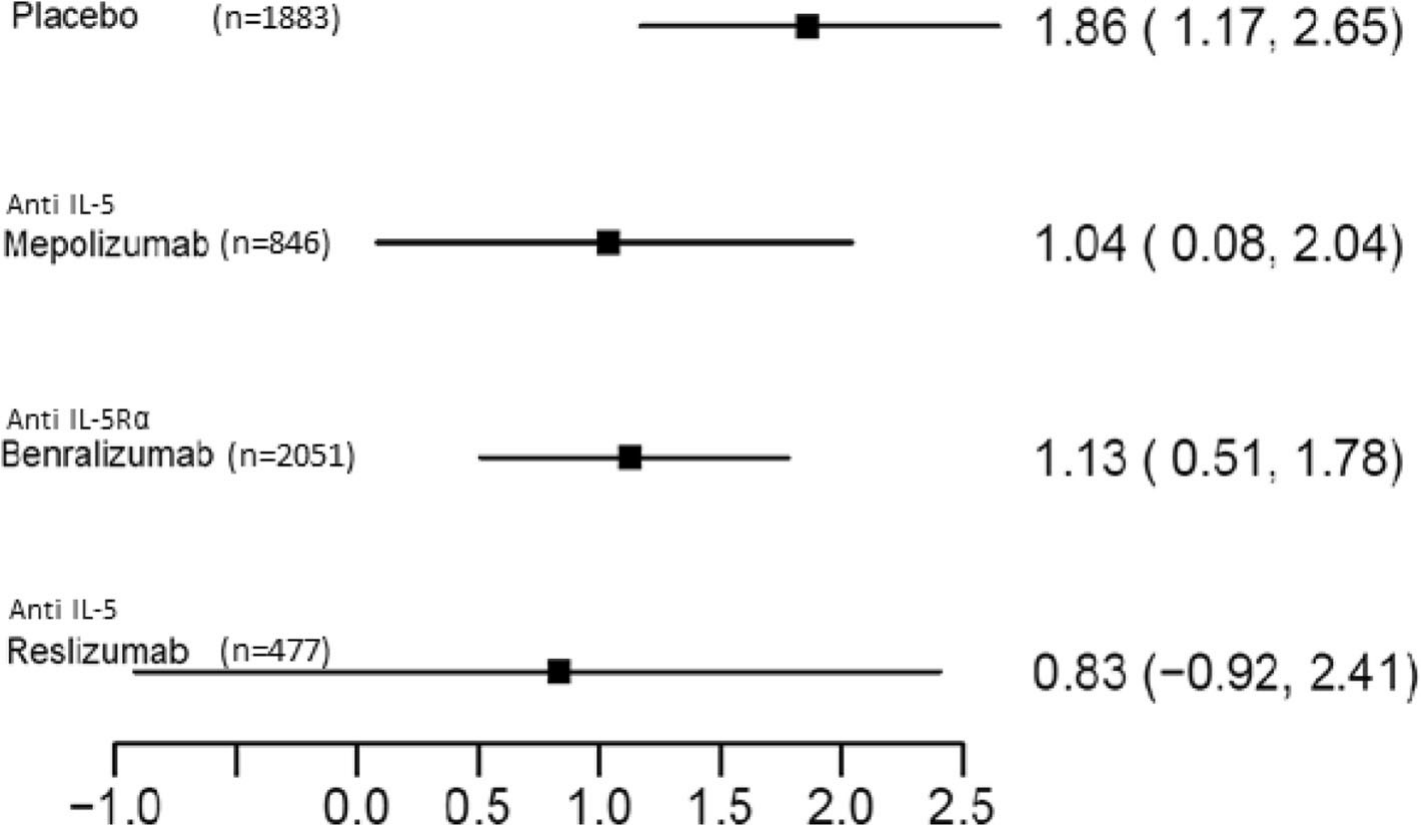
Exacerbation rates when treated with biologics acting on the interleukin 5 pathway [15, 36–42]. Median Annualized Exacerbation rate (95% CI). Forest plot of the exacerbation rates among the placebo and IL-5 agent arms

**Fig. 4:**
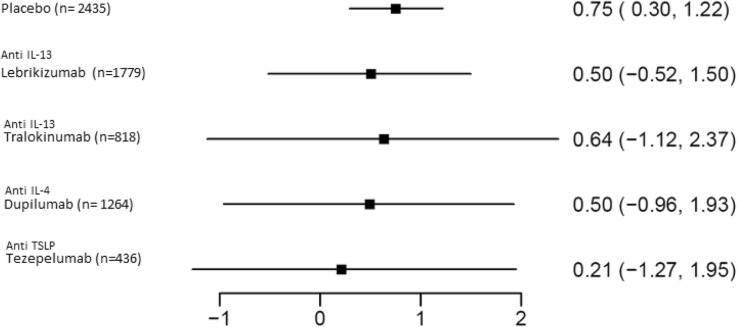
Exacerbation rates when treated with biologics not targeting the interleukin 5 pathway [13, 19, 33–35]. Median Annualized Exacerbation rate (95% CI). Forest plot showing the exacerbation rates achieved with different agents targeting non-IL-5 pathways. All had wide confidence intervals, and no statistically significant effects against placebo were detected

A network meta-analysis was performed to evaluate effect differences of the monoclonal antibodies on annualized exacerbation rates. All trials on the seven monoclonal antibodies having exacerbation rate as a primary outcome were added to the meta-analysis. Mepolizumab and benralizumab were the most investigated in the included studies. None of the included monoclonal antibodies demonstrated statistically significant effect differences on the exacerbations rate compared to placebo. (Table 2) In addition, the network meta-analysis revealed no superiority of any included biological on exacerbation rate in the indirect head to head comparisons. (Table 2) Percentage of studies with low risk of bias included for every drug is outlined in Table 3. The summary of effect sizes and confidence intervals shown in Additional file 1: Fig. S1 demonstrates the highest median exacerbation rate among the pooled placebo group and the lowest among the tezepelumab-treated arms. However, the effect estimate of the tezepelumab-treated arm had also the widest surrounding confidence interval highlighting the high uncertainty on the estimate itself and indicating the lack of power to support significant improvement compared to placebo. A downward trend in the exacerbation rate by study year (between 2012 and 2018) was observed. Sub-group analyses were conducted based on the mechanism of action. The pooled placebo group of the seven studies with drugs acting on the IL-5 pathway (mepolizumab, benralizumab and reslizumab) had a higher mean exacerbation rate compared to the pooled placebo group of trials conducted with other agents. (Figs. 3 and 4) Only the benralizumab arm was sufficiently powered (*n* = 2051) to demonstrate a significantly decreased exacerbation rate of − 0.730 (95% confidence interval − 1.490, − 0.051) compared to placebo in this subgroup analysis. In the second subgroup (not acting on IL-5 pathway), no single agent was sufficiently powered to show significant superiority compared to placebo.

**Table 2:** Effect differences (95% confidence interval) between treatments on exacerbation rate [13, 15, 19, 33–42]

Treatment	Placebo	Benralizumab	Lebrikizumab	Dupilumab	Mepolizumab	Tralokinumab	Reslizumab	Tezepelumab
Placebo		0.485 (− 0.132, 1.080)	0.903 (− 0.278, 2.380	0.903 (− 0.571, 2.570)	0.485 (− 0.797, 1.750)	0.755 (− 0.933, 2.640)	0.589 (− 1.100, 2.280)	1.170 (− 0.678, 3.150)
Benralizumab	−0.485 (− 1.080, 0.132)		0.445 (− 0.850, 1.870)	0.439 (− 1.120, 2.070)	−0.019 (− 1.300, 1.380)	0.283 (− 1.490, 2.130)	0.100 (− 1.620, 1.870)	0.697 (− 1.170, 2.670)
Lebrikizumab	−0.903 (− 2.380, 0.278)	−0.445 (− 1.870, 0.850)		0.006 (− 1.950, 1.760)	− 0.483 (− 2.110, 1.280)	−0.172 (− 2.180, 1.920)	−0.361 (− 2.390, 1.720)	0.258 (− 1.950, 2.460)
Dupilumab	−0.903 (− 2.570, 0.571)	−0.439 (− 2.070, 1.120)	−0.006 (− 1.760, 1.950)		− 0.472 (− 2.330, 1.530)	−0.164 (− 2.390, 2.160)	−0.347 (− 2.430, 1.740)	0.258 (− 2.070, 2.760)
Mepolizumab	−0.485 (− 1.750, 0.797)	0.019 (− 1.380, 1.300)	0.483 (− 1.280, 2.110)	0.472 (− 1.530, 2.330)		0.317 (− 1.750, 2.320)	0.122 (− 1.980, 2.150)	0.709 (− 1.580, 2.980)
Tralokinumab	−0.755 (− 2.640, 0.933)	− 0.283 (− 2.130, 1.490)	0.172 (− 1.920, 2.180)	0.164 (− 2.160, 2.390)	−0.317 (− 2.320, 1.750)		− 0.188 (− 2.620, 2.230)	0.408 (− 2.000, 2.970)
Reslizumab	− 0.589 (− 2.280, 1.100)	−0.100 (− 1.870, 1.620)	0.361 (− 1.720, 2.390)	0.347 (− 1.740, 2.430)	−0.122 (− 2.150, 1.980)	0.188 (− 2.230, 2.620)		0.604 (− 1.810, 3.090)
Tezepelumab	−1.170 (− 3.150, 0.678)	− 0.697 (− 2.670,1.170)	−0.258 (− 2.460, 1.950)	−0.258 (− 2.760,2.070)	−0.709 (− 2.980, 1.580)	−0.408 (− 2.970, 2.000)	−0.604 (− 3.090, 1.810)	

Table showing the effect differences detected between all agents compared to placebo, all effect differences were not statistically significant and had wide confidence intervals, and therefore no agents could prove superior to another. The studies on IL-5 pathway agents included patients with a relatively higher number of exacerbations, due to selecting patients with a history of exacerbations and documented eosinophilia, which should be taken into account when interpreting their compared effects. No agent could prove superior to another in these indirect head to head comparisons

**Table 3:** Percentage of low risk of bias studies per treatment arm in the meta-analysis on exacerbation rate [13, 15, 19, 33–42]

Treatment	Percentage
Benralizumab	60% (3/5)
Lebrikizumab	50% (1/2)
Dupilumab	0% (0/1)
Mepolizumab	50% (1/2)
Tralokinumab	100% (1/1)
Reslizumab	100% (1/1)
Tezepelumab	100% (1/1)

Table outlining the risk of bias for different studies included in the meta-analysis, all agents had at least 50% of low-risk studies included, except dupilumab

## Discussion

We retrieved a total of 30 randomized placebo-controlled clinical trials investigating the efficacy of these biologics. All trials were conducted between 2007 and 2018. Most trials (29/30) were industry sponsored, resulting in possible bias. Only 50% of the trials were deemed to be of a low-risk of bias, while 6 were assessed to be at an increased risk. The trials mostly included patients with moderate to severe asthma proven by a history of exacerbations or based on blood eosinophils level. Most trials used either exacerbation rates, FEV1 change or symptom scoring as a primary end-point. Mepolizumab, reslizumab and benralizumab have all been shown to reduce exacerbation rates for patients with moderate to severe asthma [15, 36–38, 42, 69]. Most trials included patients based on their history of exacerbations and blood eosinophilia. Reduction in exacerbation rates ranged from 40% (benralizumab) to 60% (reslizumab). Effects on quality of life varied by agent, study design and drug dose. These results show the potential clinical effects of blocking the IL-5 pathway in moderate to severe asthma.

In contrast, lebrikizumab and tralokinumab, both acting on the IL-13 pathway, have shown less consistent effects on exacerbation rates. Hanania et al. demonstrated the inability of lebrikizumab to consistently reduce exacerbation rates [34], this was also confirmed for tralokinumab by a phase II and a phase III trial [35, 59]. GSK679586, also acting on the IL-13 pathway, was only investigated by a phase II trial. It also had no effect on exacerbation rates, lung function, symptom control and health-related quality of life [62]. This may reflect the lack of clinical effect of targeting the IL-13 pathway alone. In a recent review, Parulekar et al. suggested that simultaneous targeting of both IL-13 and IL-4 pathways may benefit patients with severe asthma [70]. This theory is supported by a dupilumab efficacy trial where exacerbations were reduced by about 50% [19]. Biweekly administered dupilumab also improved lung function, symptom control and health-related quality of life [19]. It is plausible that in coming updates of leading guidelines dupilumab will be recommended as potential add-on treatment in severe asthma [19]. MEDI-528, acting on IL-9, was investigated by a phase II trial where exacerbation rates, lung function, symptom control and health-related quality of life are not affected by this intervention [20]. Tezepelumab efficacy was also only investigated by a phase II trial, exacerbation rates were decreased with about 60 to 80% and lung function is improved irrespective of the administered dose. Symptom control was improved by medium and high dose tezepelumab. Only the high dose improved the health-related quality of life [13]. Those results suggest that TSLP may also be an important drug target in asthma. Phase III trials confirming or disproving the efficacy of tezepelumab are still awaited [13].

Our network meta-analysis demonstrated that none of the studied monoclonal antibodies showed statistically significant improvement of the exacerbation rate compared to the pooled placebo, nor was any treatment arm superior in the indirect head to head comparisons. Most studies were on mepolizumab or benralizumab. Studies on benralizumab and lebrikizumab included the highest number of patients and were therefore most powered to approach statistical significance for the modest improvement on exacerbations compared to placebo. The variation between the mean rates of exacerbation rates between the pooled placebo groups and treatment arms of IL-5 pathway versus other biologics may be due to different inclusion criteria. It is also worth noting that among the included agents, only dupilumab and the three IL-5 pathway agents (mepolizumab, reslizumab and benralizumab) are currently FDA approved for severe asthma. Mepolizumab, benralizumab and reslizumab trials mostly included subjects based on previous exacerbations and high number of eosinophils. Conversely, most trials of other biologics selected subjects based only on previous exacerbations. Effect differences in our analyses should therefore be interpreted in light of that difference. Since benralizumab demonstrated statistically significant reduction in the exacerbation rate compared to placebo in this analysis, our results emphasize that adequate phenotyping characterizing the underlying endotype is key for agents targeting IL-5 to demonstrate their efficacy. It should be of note that further high quality trials on the included treatments and direct head to head comparisons between the biologic agents may be needed to fully compare between the treatment modalities independent of patient selection differences. The difference in the risk of bias for the studies included in the meta-analysis is also of note. For example, benralizumab had the lowest percentage of included studies with low risk of bias, which may have affected the positive significant effect seen in the IL-5 pathway sub-analysis. In an earlier review of the evidence by the Institute of Clinical and Economic Review (ICER), mepolizumab was considered to be of modest benefit in terms of reducing exacerbations and improving quality of life [71]. Evidence regarding benralizumab, reslizumab and dupilumab efficacy was considered of moderate certainty, and the possibility that biologics effects were comparable to placebo could not be ruled out [71].

Most trials included patients based on the number of exacerbations in the previous year, but results were more consistent where patients had higher levels of eosinophilia [37, 53, 54]. However, reductions in eosinophil levels cannot infer clinical effects, as proven by lebrikizumab and tralokinumab trials [34, 35, 59]. This suggests that eosinophil levels may be used as a biomarker to select patients predicted to benefit the most from treatment. It is difficult however to use its post-treatment as a marker of clinical efficacy. This was recently tested by Kelly et al. who proved that T cells retain some functionality after 750 mg doses of mepolizumab even when median values of circulating eosinophils dropped by 75% [72]. Therefore a reduction in eosinophil numbers cannot infer clinical efficacy, and clinical outcomes must be used to evaluate efficacy.

Exposure to allergens may initiate naive T-lymphocytes differentiation towards type 2 helper T-lymphocytes (T_H_2 cells) in genetically susceptible individuals [73]. The allergens inhalation elicits epithelial attraction of dendritic cells, as well as epithelial production of thymic stromal lymphopoietin (TSLP), interleukin (IL)-33 and IL-25 (pro-T_H_2 co-activating cytokines). The antigen-presenting dendritic cells migrate to the lymph nodes, where IL-4 provides the initiating stimulus for the T_H_ polarization towards T_H_2 cells. T_H_2 cells produce their typical T_H_2-associated cytokines: IL-4, IL-5, IL-9 and IL-13 [67]. Additionally, innate type 2 lymphoid cells (ILC2) are a potent source of IL-5 and IL-13. ILC2 cells may be activated by non-allergenic or infectious stimuli [74]. Increased ILC2 numbers were associated with severe asthma with persistent eosinophilia [75, 76]. IL-33 (ILC2 activator, besides TSLP and IL-25)) was also associated to airway remodeling in steroid resistant asthma [77]. IL-4 and IL-13 share a common receptor: IL-4Rα. Both cytokines are powerful mediators of type 2 immunity. IL-4 is the key factor in the T_H_2 type response: it guides the naive T cell differentiation to the T_H_2 subtype [63]. IL-4 also steers the isotype switch of B cells towards immunoglobulin (Ig) E. The main function of IL-13 consists mediating goblet cell hyperplasia and smooth muscle contractility. In addition, IL-13 has an additional role in isotype class switching and IgE production. IgE is the hallmark of allergic sensitization. It has the potential to activate mast cells and basophils. IL-5 is the key factor in the maturation and survival of eosinophils. It is suggested that IL-9 mediates the mast-cell component of the allergic reaction [47, 56]. Type 2-associated asthma is characterized by eosinophilic airway inflammation [78]. Blood eosinophilia and FENO were the most robust markers for this inflammation [79].

The safety profile and long term effects of those biologic agents are also yet to be established. The adverse reactions noted in the trials were generally limited. However, the trials may have been too short or underpowered to detect rare serious adverse events. Given possible seasonal effects on asthma, one-year long trials are preferred instead of shorter ones. An important example is daclizumab, removed from the market in 2018 due to cases of encephalitis [80]. Busse et al. detected in 2008 five adverse events in the treatment group, but a causal relationship could not be established [68]. Two open-label extensions of mepolizumab and reslizumab trials have been published [23, 24]. Overall, the drugs had a favorable side effects profile, however, there was an increased percentage of adverse events in the treatment groups and 7% of reslizumab users experienced a serious adverse event [24]. This highlights the importance of open-label extensions, and rigorous pharmacovigilance when using the new biologic agents.

It is worth noting that there is a dissociation between improved asthma outcomes and patient reported outcomes. Having improved lung function or less exacerbations does not automatically lead to better symptom control or health-related quality of life. For example, lebrikizumab improved lung function but ACQ and AQLQ were not affected. Equal observations were made for tezepelumab. Its effect on lung function and exacerbation rate was dose-independent. In contrast, ACQ only improved in medium and high dose tezepelumab, and AQLQ only in the high dose group. The anti-IL-5 and anti-IL-5R biologics all significantly reduced the number of exacerbations. However, the results about ACQ and AQLQ were less consistent.

Biologics are considered to be expensive, emphasizing the importance of confirming the diagnosis and assessing modifiable factors, therapy compliance and inhaler technique before their initiation. However, it remains vital to invest in new and innovative therapeutic agents. More adequate asthma treatment results in better symptom control and less exacerbations and therefore with taking less time of work, less emergency department visits and hospitalizations. In doing so, targeted therapeutics may be cost-effective [81]. In the ICER review, cost-effectiveness analysis of biologic therapies in asthma (including omalizumab, reslizumab, mepolizumab, benralizumab and dupilumab) estimates did not meet commonly-cited cost-effectiveness thresholds [71]. The subpopulations with ≥300 eosinophil count did not change the results substantially from the base-case as well.

Some important topics are not discussed in this review. Little real-world evidence of biologics’ effectiveness exists. The effect of biologics on top of controller therapy was undiscussed. Future research should further investigate whether controller therapy can be reduced after disease control with biologics and whether treatment with biologics could be stopped after a certain duration time.

This review primarily points out the major findings due to difficulties comparing different trials with identical biologics, or comparing between different biologics. This is caused by diversity in administered doses, routes of administration, inclusion criteria and primary outcomes. Clinical efficacy should be interpreted in light of the selection criteria used in the trials. For example, reslizumab seemed to obtain greater reductions in exacerbation rates compared to mepolizumab and benralizumab. However, this result cannot be generalized as the leading reslizumab trial only selected patients with higher baseline blood eosinophil counts, less exacerbations in the previous year and lower controller treatment compared to mepolizumab and benralizumab. Another example is lebrikizumab, which only improved lung function in patients with high serum periostin, a variable that is not tested in other trials, therefore comparison with other biologics is nearly impossible.

These examples demonstrate the need for further refinement of current described endotypes. It is clear that some biologics are more efficacious when given to patients with elevated levels of certain biomarkers but the threshold of these biomarkers that results in significant improvements is not specified for any of the biologics. Furthermore, head to head trials between different biologics are necessary to make better assessments about which biologic may be the preferred therapeutic for a particular patient. Some agents currently in early development phases were also not included, which may have an important impact on asthma in the future [82].

## Conclusion

In conclusion, monoclonal antibodies are promising therapeutics for the treatment of severe, persistent asthma. Several phase III trials demonstrated the efficacy of mepolizumab, reslizumab and benralizumab and the efficacy of dupilumab has been recently confirmed. Dupilumab will potentially be added to the recommended biologics for the treatment of severe asthma in near future. Phase III trials that confirm or disprove the efficacy of tezepelumab are awaited. Lebrikizumab, tralokinumab, GSK679586 and MEDI-528 have no or inferior effects on asthma outcome. Daclizumab improved FEV1, but was later removed from the market due to side effects. In general, the lack of well-defined endotypes is a major hurdle to the interpretation and implementation of trial results. Response is defined by observable features and biomarkers, but no cut-off values or point of care testing are currently available. Therefore, there are diverging inclusion criteria among the several trials. Thus, endotypes need to be further refined, and selecting severe asthma patients based on their eosinophilia and number of exacerbation appears to be a sound strategy and an important precision medicine opportunity. Head to head trials between different biologics may be necessary to determine the best therapeutic option for a particular patient. To estimate and determine long-term effects, patients treated with monoclonal antibodies should be followed up long-term.

## Additional file

Additional file 1: Online Supplement.Methods. **Table S1.** Agents targeting interleukin-5 in the treatment of asthma. (Table 1 continues on the next page). **Table S2.** Agents targeting interleukin-13 in the treatment of asthma. **Table S3.** Agents targeting both interleukin-4 and interleukin-13 in the treatment of asthma. **Table S4.** Agents targeting interleukin-9 in the treatment of asthma. **Table S5.** Agents targeting thymic stromal lymphopoietin in the treatment of asthma. **Table S6.** Agents targeting CD25. **Figure S1.** Exacerbation rates among the different treatment arms, ordered by number of subjects treated. (DOCX 285 kb)

## Data Availability

Not applicable

## Abbreviations

ACQ-7: Asthma control questionnaire 7
AQLQ: Asthma Quality of Life Questionnaire
CCR3: Chemokine receptor type 3
ER: Emergency Room
FeNO: Fraction of exhaled nitric oxide
FEV_1_: Forced expiratory volume in one second
FVC: Forced Vital Capacity
GINA: Global Initiative of Asthma
ICER: Institute of Clinical and Economic Review
ICS: Inhaled Corticosteroids
IgE: Immunoglobulin E
IL-13: Interleukin 13
IL-25: Interleukin 25
IL-33: Interleukin 33
IL-4: Interleukin 4
IL-4Rα: Interleukin 4 Receptor Alpha
IL-5: Interleukin 5
IL-5Rα: Interleukin-5 receptor α
IL-9: Interleukin 9
ILC2: Innate type 2 lymphoid cells
IV: Intravenous
LABA: long acting beta-agonist
PEF: Peak Expiratory Flow
PGD2: Prostaglandin D2
SC: Subcutaneous
T_H_2-high: Asthma endotype characterized by high Type 2 inflammation
TSLP: Thymic stromal lymphopoietin
