# Supplementary material for: Monoclonal antibodies in type 2 asthma: a systematic review and network meta-analysis
Source: Respir Res. 2019 Aug 8;20:179. doi: 10.1186/s12931-019-1138-3 (PMC6688359; doi:10.1186/s12931-019-1138-3)
Supplement: Supplementary file 1 — Online Supplement.Methods. Table S1. Agents targeting interleukin-5 in the treatment of asthma. (Table 1 continues on the next page). Table S2. Agents targeting interleukin-13 in the treatment of asthma. Table S3. Agents targeting both interleukin-4 and interleukin-13 in the treatment of asthma. Table S4. Agents targeting interleukin-9 in the treatment of asthma. Table S5. Agents targeting thymic stromal lymphopoietin in the treatment of asthma. Table S6. Agents targeting CD25. Figure S1. Exacerbation rates among the different treatment arms, ordered by number of subjects treated. (DOCX 285 kb) [file 12931_2019_1138_MOESM1_ESM.docx]

**Online Supplement**

**1-Methods:**

**1.1-Search Terms:**
The search terms used in both databases were: ‘asthma’, ‘interleukin-5’, ‘Mepolizumab’, ‘Reslizumab’, ‘Benralizumab’, ‘interleukin-4’, ‘interleukin-13’, ‘Lebrikizumab’, ‘Tralokinumab’, ‘Dupilumab’, ‘Pitrakinra’, ‘interleukin-9’, ‘thymic stromal lymphopoietin’, ‘Tezepelumab’.

**1.2-Data Extraction**

The following data was extracted from included studies in a table: author, year, journal, inclusion criteria, main outcome, drug, administration frequency, dose, mean effect on the main and secondary outcomes, standard deviations or confidence intervals and number of subjects involved.

**1.3-Statistical Analysis**

The pcnetmeta package uses Markov chain Monte Carlo (MCMC) algorithms to estimate parameters on the R platform through JAGS, a program for analyzing Bayesian hierarchical models.[1, 2]

The exacerbation rates (means with standard deviations) among the placebo and treatment groups were used to calculate effect differences and 95% CI. Missing standard deviation values were imputed using the package metagear in R.[3] For studies that reported only confidence intervals standard deviation was calculated based on the upper and lowers ends of the confidence interval and the number of subjects involved. Where studies did not report confidence intervals or standard deviations for the mean, standard deviation was inferred from confidence intervals of reduction percentages reported in the studies.

**2-Results of additional endpoints in the different agents’ trials:**

**2.1-Mepolizumab
Blood eosinophils:**

Mepolizumab reduced blood eosinophil counts, irrespective of the dose and route of administration. IV administration reduced eosinophils by 78% to 88% compared to placebo, 86% reduction was achieved with SC administration. Improvements were obtained by the first on-treatment assessment and maintained throughout the studies. Blood eosinophil counts started to rise again after discontinuation of the study drug.[4-6] The effect of mepolizumab on sputum eosinophil

counts is only investigated for IV mepolizumab. Low and medium dose (75 mg and 250 mg) did not alter sputum eosinophilia. High dose mepolizumab (750 mg) reduced the sputum eosinophil counts significantly by 73% to 88% compared to placebo.[5, 6]

**FEV1 improvement:**

The DREAM-trial(Phase II) demonstrated negligible improvements in prebronchodilator FEV_1_.[5] In contrast MENSA-trial (Phase III) demonstrated improved lung function following mepolizumab treatment.[4] The mean change from baseline in prebronchodilator FEV_1_ was 0.098 L and 0.1 L compared to placebo after 32 weeks of SC and IV mepolizumab, respectively.[4] Similarly, DREAM did not show improvements in symptom control.[5] The MENSA-trial indicated improved asthma symptom control, with reductions in ACQ-5 at week 32 with -0.44 points and -0.42 points for SC and IV mepolizumab, respectively.[4] The DREAM-trial resulted in a non-significant improvement in health-related quality of life compared to another phase II trial where AQLQ was 0.35 points higher compared to placebo.[5, 6] The MENSA-trial used another questionnaire to evaluate quality of life: the St. Georges Respiratory Questionnaire (SGRQ), which was significantly improved by mepolizumab treatment.[4] Chupp et al. also showed significant reductions in SGRQ at week 24.[7] The study compared 274 subjects on mepolizumab 100mg SC to 277 controls, improvement compared to placebo exceeded the minimal clinically important difference -defined as a four point reduction in the SGRQ score- from week four onwards.[7]

**2.2-Reslizumab:
Blood eosinophils:**In the eosinophil high population (blood eosinophils ≥400 cells/µL or sputum ≥3%), blood eosinophilia decreased with 420 up to 494 cells/µL at the end of treatment compared to placebo. However, the counts rose again at the follow up visit several months after the last administration.[8-10] In a population not selected based on eosinophil level, counts were reduced by 260 cells/µL compared to placebo after 16 weeks of reslizumab treatment.[11] One trial also tested the effect on sputum eosinophilia. The reslizumab group had 95.4% less sputum eosinophils compared to baseline, compared to 37% in the placebo group.[10]

**FEV1 improvement:**

In the eosinophilic population, the mean change from baseline in prebronchodilator FEV_1_ by reslizumab versus placebo ranged from 0.090 L to 0.240 L after a 12 to 52-week reslizumab treatment [8-10]. The results are in line with an improvement in 52 weeks.[12] In a population not selected for a certain eosinophil level, FEV_1_ was not significantly improved. In contrast, the subgroup of reslizumab-treated patients with blood eosinophil counts ≥400/µL showed large improvements in FEV_1_ (0.270 L) in comparison with placebo. However, this study was not powered for subgroup analyses [11]. Similar trends were observed for FVC: rapid and maintained improvements in eosinophil-high patients [8, 10, 12], but without significant improvement in a population with patients with and without eosinophilia ≥400/µL.[11]

**Symptom control and quality of life:**

Improved symptom control by reslizumab treatment was evident across all phase III trials. The decline in ACQ-5, ACQ-6 or ACQ-7 relative to placebo ranged from -0.20 points to -0.36 points after a 16 to 52-week reslizumab treatment. The older phase II study did not show better symptom control.[10] The effect of reslizumab on health-related quality of life was only investigated in a patient population with baseline blood eosinophilia ≥400 cells/µL. Improvements in AQLQ were rapid and sustained, ranging from 0.23 points to 0.36 points compared to placebo by the end of the trials**.** The proportion of patients achieving a minimally important difference of 0.5 points improvement was higher in reslizumab (64% to 74%) than placebo (48% to 65%).[8, 9, 12]

**2.3-Benralizumab
Blood eosinophils:**Baseline eosinophil counts ranging from 440 to 480 cells/µL were reduced to a median of 0 cells/µL by the first on-treatment assessment at week 4. The cell counts then remain stable throughout the trial. Similar results were seen for patients with baseline eosinophil counts <300 cells/µL. These results were independent from benralizumab dose and the length of the administration interval.[13, 14] One phase II trial that selected possible responders by sputum eosinophilia ≥2% and also included patients with medium dose ICS + LABA revealed similar results.[15]

**FEV1 improvement:**

Benralizumab treated patients did better in terms of lung function according ot SIROCCO, CALIMA and BISE trials.[13, 14, 16] The mean change from baseline in prebronchodilator FEV_1_ was 0.106 L to 0.125 L in the Q4W-arm and 0.116 L to 0.159 L in the Q8W-arm, compared to placebo. The improvements were present within the first 4 weeks of treatment and remained for the trial duration. Among these patients, those who had at least 3 exacerbations in the previous year would benefit benralizumab the most, especially in the Q8W-arm. FEV_1_ did not improve in benralizumab-treated asthma patients with <300 blood eosinophils/µL or with ≥300 blood eosinophils/µL and treated with medium-dosage ICS + LABA as maintenance therapy.[13, 14] BISE showed an 80 ml FEV_1_ increase, but results were statistically inconsistent (CI 0-150).[16] Park et al., showed significantly improved morning PEF. Changes range from 15% to 16.2% by a 52 week-during 2 mg, 20 mg or 100 mg benralizumab treatment, compared to a 9.4% improvement in the placebo arm.[15]

**Symptom control and quality of life:**

the SIROCCO trial showed a significant decrease in ACQ-6 score (‑0.29 points) for the Q8W regimen compared to placebo. However, no significant improvements were observed for the Q4W regimen. Conversely, CALIMA trial, sowed improved ACQ-6 scores relative to baseline compared to placebo for the Q4W regimen (-0.19 points) and the Q8W regimen (-0.5 points). Both the SIROCCO and the CALIMA noted a significant improvement in quality of life in their target population by the benralizumab Q8W regimen (0.24 points to 0.30 points), but not in the Q4W regimen. Those patients with at least 3 exacerbations in the previous year benefited from benralizumab treatment most, with the greatest improvements in ACQ-6 as well as AQLQ compared to placebo.[13, 14]

**2.4-Lebrikizumab**
**FEV1 improvement:**

Lebrikizumab has a generally beneficial effect on lung function, as noted by prebronchodilator FEV_1_ [17, 18]. However, improvements in FEV_1_ are only considered to be significant in serum periostin-high (>50 ng/mL) patients treated with lebrikizumab 125 mg or 250 mg. In these patients, prebronchodilator FEV_1_ increased by 10.7% and 8.2% to 10.1%, respectively.[17, 18] In LAVOLTA I and II, both periostin high and low patients showed better efficacy and treatment by biomarker interaction was strongest for eosinophils.[19] In contrast, LUTE and VERSE trials as well as Corren et al. suggest that treatment effects differed by periostin level.[17, 18] Changes in FEV_1_ were evident within one week of treatment and maximal improvements are reached by week 4.[17, 18] Non-significant trends towards improvements in morning PEF of 20.92 L/min (95% CI 2.00 L/min to 39.84 L/min) in lebrikizumab 250 mg treated patients with low serum periostin levels.[17, 20] Lebrikizumab reduced FeNO by 19% to 49%, compared to a 10% to 11% increase in the placebo groups.[18, 20]

**Symptom control and quality of life:**

No clinically meaningful improvement in symptom control or health related quality of life, as assessed by ACQ or AQLQ was detected in the four trials [17-20].

**2.5-Dupilumab:
FENO improvement:**Dupilumab inhibits both IL-13 and IL-4, therefore reduced FeNO and IgE counts are expected. The decline in FeNO is demonstrated to range from 16.4% to 40.3% compared to placebo in a patient population that is not selected for a certain baseline eosinophil level. The decreases are greatest for dupilumab given every 2 weeks and in the subpopulation with elevated eosinophil counts. Patients with lower baseline eosinophil counts also obtain reduced FeNO, although the reduction is less substantial. Both the 200 mg and 300 mg dose, as well as the weekly, biweekly and monthly dupilumab administrations succeed to reduce FeNO. Dupilumab treatment also reduces IgE levels in both a selected and non-selected patient population for a certain baseline eosinophil level, irrespective of the administered dose and the length of the administration interval.[21-23]

**FEV1 improvement:**

Dupilumab induces a rapid improved lung function, and maintains the improvements during the complete intervention period. If patients are not selected for blood or sputum eosinophilia, change in FEV_1_ from baseline is 0,140 L to 0,200 L and 0,130 L to 0,160 L after 12 weeks of biweekly treatment with 200 mg and 300 mg dupilumab, respectively. Patients with elevated eosinophil counts (≥300 cells/µL in blood or ≥3% in sputum) have greatest treatment benefits.[21, 23] Patients with lower eosinophil levels also benefit dupilumab treatment, although improvements in FEV_1_ are smaller [21, 23]. Next to blood eosinophilia, FeNO (<25 ppb or ≥25 ppb) is another useful predictor of treatment response to dupilumab. Monthly administration provides less improvements FEV_1_, especially in patients with blood eosinophil levels <300/µL.[23] Dupilumab would influence morning PEF in a positive way, although results for evening PEF are contradictory.[21, 22]

**Symptom control and quality of life:**

Symptom control, as quantified by ACQ-5, is significantly improved by weekly or biweekly dupilumab administration (overall population: -0.30 points to -0.35 points compared to placebo; blood eosinophilia ≥300/µL: -0.42 points to -0.73 points). There are also noted improvements in the AQLQ by biweekly and monthly dupilumab administration (overall population: 0.31 points to 0.36 points; blood eosinophilia ≥300/µL: 0.43 points to 0.78 points). The only exception is the monthly administration of dupilumab 200 mg, which does not affect health-related quality of life.[21-23]

**2.6-Tezepelumab:**

**Blood eosinophils and FENO levels:**

Blood eosinophil counts and FeNO levels were rapidly and persistently decreased by tezepelumab treatment in all intervention groups. Serum IgE decreased over time.[24] Additionally, tezepelumab successfully improved lung function. Mean change from baseline in prebronchodilator FEV_1_ after one year of treatment was 0.120 L in the low-dose group (70 mg every 4 weeks), 0.110 L in the medium-dose group (210 mg every 4 weeks) and 0.150 L in the high-dose group (280 mg every 2 weeks). FEV_1_ improved as early as the first post-treatment assessment. The improvement was maintained over the trial duration.[24]

**Symptom control and quality of life:**

Medium- and high-dose tezepelumab treatment improved asthma symptom control. Reductions in ACQ-6 ranged from -0.27 to -0.33 points compared to placebo. Improvements by low-dose tezepelumab were negligible. Only the high-dose group benefited the study drug in terms of quality of life: AQLQ improves with 0.38 points compared to placebo.[24]

**3-Safety Results:**

**3.1-Side Effects of anti-IL5 monoclonal antibodies(Mepolizumab, Benralizumab and Reslizumab:**

Anti-IL5 monoclonal antibodies were generally well tolerated. Percentage of patients reporting at least one adverse event varied by drug (mepolizumab: 78% to 86% of patients, reslizumab 55% to 80% and benralizumab 72% to 96%). Injection site reactions were less than 1% for reslizumab, 4 to 12% for mepolizumab, while benralizumab had a higher incidence (2 to 36%). Anti-drug antibodies (ADA) were detected for the three drugs, with different percentages (mepolizumab 0 to 9%, reslizumab w to 11%, benralizumab 8.3% to 15%). No serious clinical effects were attributed to ADAs. No deaths were related to the study drugs, and serious adverse events were usually rare. 3 anaphylactic reactions were attributed to reslizumab, so close monitoring in the immediate post-infusion period is essential. Serious adverse events attributed to the three drugs were generally rare. Reported adverse events were usually mild to moderate, headaches and upper respiratory tract infections were reported for the three agents.

**3.2-Side Effects of Lebrikizumab:**

Lebrikizumab is generally well tolerated: 67.7% to 80.4% of patients in the lebrikizumab groups had at least one AE, compared to 67.3% to 78.6% in the placebo groups. Most were mild to moderate in severity. Noteworthy, musculoskeletal side effects were more frequent in the lebrikizumab group compared to placebo (13.2% versus 5.4%) as well as injection site reactions (11.1% to 23.4% versus 5.8% to 6.0%). A SAE considered to be related to the study drug was sarcoidosis. Three to 3.8% of the patients treated with lebrikizumab withdrew because of AE. No anaphylactic reactions, reported neoplasms related to the study drug and no deaths are reported. ADA responses are observed, but they are not considered to be associated with any AE.[17, 18, 20] Peripheral blood eosinophil counts slightly increase by lebrikizumab treatment. This may be due to decreased migration from eosinophils from blood to the airways in response to the inhibition of IL-13 associated chemokines.[17, 18]

**3.3-Side Effects of Tralokinumab**

Tralokinumab has an acceptable safety profile. Incidence of adverse events was similar in treatment and placebo groups among the studies. Frequently reported AE are headache and nasopharyngitis, urinary related AE and diarrhea (only in subjects receiving the highest dose.). No SAE were linked to the tralokinumab treatment. Injection site reactions (6.3% to 7.8%) and a slight increase in baseline blood eosinophil counts were reported in the tralokinumab group in Piper et al.[25]

**3.4-Side Effects of GSK679586:**

53% of the patients in the GSK679586 intervention arm reported an adverse event compared to 49% in the placebo arm. Two patients who discontinued early had adverse events considered to be related to the study treatment: 1 patient in the placebo group (fatigue) and 1 patient in the GSK679586 group (mild gastrointestinal disturbance and parasite positive stool sample). Two of the 15 SAE were assessed as related to the study drug: lethargy and supraventricular extra-systoles, but they did not lead to early discontinuation. Two GSK679586 treated patients presented with ADA, but no neutralizing antibodies are determined and the safety profiles are unaffected.[26]

**3.5-Side Effects of GSK679586:**

Similar proportions of adverse events were reported across the dupilumab and the placebo groups (75% to 83% for dupilumab versus 75% to 83% for placebo), usually nonspecific and of mild-to-moderate intensity. Frequently reported AE are upper respiratory tract infections, headache, nausea and a transient increased blood eosinophilia. Most of the elevations in eosinophil counts did not have any clinical consequences, although hypereosinophilia was exceptionally associated with adverse events as worsening of hypereosinophilia and chronic eosinophilic pneumonia. Injection site reactions occurred in 15.2% of the dupilumab treated patients. One dupilumab treated patient was reported with angioedema. Conjunctivitis is observed in similar proportions across the dupilumab and the placebo groups (2.3% dupilumab versus 3.3% placebo), in contrast to studies of dupilumab treatment for atopic dermatitis. Dupilumab does not increase the risk of bacterial or opportunistic herpes viral infections. The incidence of serious adverse events is similar between both treatment groups. No deaths were considered to be related to the study drug. ADA are reported in 2.1% to 4.2% of dupilumab treated patients, but without any clinical implication.[21-23]

**3.6-Side Effects of MEDI-528:**

MEDI-528 is not associated with major safety concerns. 76.5% to 85% of the MEDI-528 group experienced at least one AE, compared to 82.8% in the placebo group. AE more frequently reported in the MEDI-528 group are asthma, upper respiratory tract infections and headache. One subject had a mild hypersensitivity reaction that resolved spontaneously the following day. 2 SAE are possibly related to the study drug: pneumonia and asthma. 6 patients discontinued the study early due to AE of which 2 are possibly related to MEDI-528: musculoskeletal pain and pain in extremity. 9% of the intervention group had ADA.[27]

**3.7-Side Effects of Tezepelumab:**

Sixty-two percent of the patients in the placebo group reported at least 1 AE vs 61.6% to 66.2% in the tezepelumab groups. Frequently reported AE were asthma, nasopharyngitis and headache. Three SAE were deemed to be related to the study drug: pneumonia, stroke and Guillain-Barré syndrome. One percent of patients in the tezepelumab group discontinued the study due to adverse events. There were no anaphylactic incidents. One patient died in the post-treatment period from a treatment related SAE (stroke).[24] ADA were reported in 0.7% to 4.9% of the tezepelumab groups, but they did not result in discontinuation.[24]

| Table 1: Targeting interleukin-5 in the treatment of asthma. (Table 1 continues on the next page) | | | |  |  |
| --- | --- | --- | --- | --- | --- |
| TARGETING INTERLEUKIN-5 | | | |  |  |
| MEPOLIZUMAB (anti-IL-5 / IV or SC) | | | |  |  |
|  | **Intervention** | **Inclusion criteria** | **Primary outcomes(reported as)** | **Effect (95%CI)** | **P value** |
|  |  |  |  |  |  |
| Ortega et al  MENSA  2014 [4]  Phase 3  N Engl J Med | 75 mg or 100 mg  Every 4 weeks  32 weeks  n=576 | Adults and children (≥12 years old); pre-bronchodilator FEV_1_ <80% (adults) and FEV_1_ <90% or FEV_1_/FVC <0.8 (adolescents); treated with fluticasone propionate ≥880 µg/day or equivalent and additional controller medication; ≥2 exacerbations treated with systemic glucocorticoids in the previous year; eosinophil blood count ≥150/µl at screening or ≥300/µl in the previous year; one or more of the following: bronchodilator response >12%, positive methacholine or mannitol challenge in the previous year, FEV_1_ variability ≥20% between 2 clinic visits | Exacerbation rate (%reduction) | 47(28-60) | P<0.001 |
| Pavord et al  DREAM  2012 [5]  Phase 3  Lancet | 75 mg, 250 mg or 750 mg  Every 4 weeks  52 weeks  n=621 | Adults and children (12-74 years old); treated with fluticasone propionate ≥880 µg/day or equivalent plus additional controller medications; ≥2 exacerbations requiring systemic corticosteroid treatment in the previous year; one or more of the following: sputum eosinophil count ≥3%, blood eosinophil count ≥300/µl, FeNO ≥50 ppb, deterioration after ≤25% corticosteroid reduction; one or more of the following: PEF variability ≥20%, bronchodilator response ≥12% and 200 mL, FEV_1_ variability ≥20% between 2 clinic visits, PC_20_ ≤8 mg/mL | Exacerbation rate (%reduction) | 48(31-61) | p<0·0001 |
| Haldar et al  2009 [6]  Phase 2  N Engl J Med | 750 mg  Every 4 weeks  50 weeks  n=61 | Adults (≥18 years old); treated with high dose corticosteroids; ≥2 exacerbations requiring rescue prednisolone in the previous year; sputum eosinophil count >3%; one or more of the following: PEF variability ≥20%, bronchodilator response ≥15%, PC_20_ ≤8 mg/mL | Exacerbation rate (relative risk) | 0.57(0.32-0.92) | P=0.02 |
| Flood-Page et al  2007 [28]  Phase 2  Am J Respir Crit Care Med | 250 mg or 750 mg  Every 4 weeks  12 weeks  n=362 | Adults (18-55 years old); pre-bronchodilator FEV_1_ 50-80% of predicted; bronchodilator response ≥12%; treated with beclomethasone dipropionate 400 - 1000 µg/day; daily symptom score ≥4/12 | Change in PEF at week 12 and 20 (mean change) | 13.49(0.71-26.27) (Mepolizumab 250mg) | 0.039 |
| Chupp et al., 2017[7] | Mepolizumab 100 mg SC or placebo, plus standard of care, every 4 weeks for 24 weeks  n=551 | Patients aged 12 years or older with severe eosinophilic asthma and a history of at least two exacerbations requiring treatment in the previous 12 months before screening despite regular use of high-dose inhaled corticosteroids plus other controller medicines. | mean change from baseline in the St George's Respiratory Questionnaire (SGRQ) total score at week 24 (treatment difference) | -7·7 (95% CI -10·5 to -4·9) | p<0·0001 |
| RESLIZUMAB (anti-IL-5 / IV) | | | |  |  |
|  | **Intervention** | **Inclusion criteria** | **Primary outcomes** | **Effect (95%CI)** | **P value** |
| Bjermer et al  2016 [8]  Phase 3  Chest | 0.3/3.0 mg/kg  Every 4 weeks  16 weeks  n=315 | Adults and children (12-75 years); bronchodilator response ≥12%; treated with fluticasone propionate ≥440 µg/day or equivalent; blood eosinophil count ≥400/µL; ACQ-7 ≥1.5 | Change in FEV_1_ at week 16 | 0.3 mg/kg: 115 mL (16-215)  3.0 mg/kg: 160 mL (60-259) | P = .0237  P = .0018 |
| Corren et al  2016 [11]  Phase 3  Chest | 3.0 mg/kg  Every 4 weeks  16 weeks  n=492 | Adults (18-65 years); bronchodilator response ≥12%; treated with fluticasone propionate ≥440 µg/day or equivalent; ACQ-7 ≥1.5 | Change in FEV_1_ at week 16(mean change) | 86 ml (SE±49) | P=0.17 |
| Castro et al  2015 [9]  Phase 3  Lancet | 3.0 mg/kg  Every 4 weeks  52 weeks  n=953 | Adults and children (12-75 years); bronchodilator response ≥12%; treated with fluticasone propionate ≥440 µg/day or equivalent; ≥1 exacerbation in the previous year; blood eosinophil count ≥400/µl; ACQ-7 ≥1.5 | Exacerbation rate | rate ratio : study 1: 0·50 (0·37-0·67] study 2: 0·41 (0·28-0·59) | p<0·0001 |
| Castro et al  2011 [10]  Phase 2  Am J Respir Crit Care Med | 3.0 mg/kg  Every 4 weeks  12 weeks  n=106 | Adults (18-75 years); bronchodilator response ≥12% or ≥20% reduction in FEV_1_ after methacholine ≤16 mg/ml; treated with fluticasone ≥880 µg/day plus ≥1 additional controller medication; sputum eosinophil count ≥3%; ACQ ≥1.5 | Change in ACQ at week 15 (difference in mean) | -0.4 | 0.054 |

| TARGETING INTERLEUKIN-5 (cont.) | | | |  |  |
| --- | --- | --- | --- | --- | --- |
| BENRALIZUMAB (anti-IL-5Rα / SC or IV) | | | |  |  |
|  | **Intervention** | **Inclusion criteria** | **Primary outcomes** | **Effect (95%CI)** | **P value** |
| Bleecker et al  SIROCCO  2016 [13]  Phase 3  Lancet | 30 mg  Every 4 weeks or every 8 weeks  48 weeks  n=1205 | Adults and children (12-75 years old); pre-bronchodilator FEV1 ≤80% (adults) or ≤90% (children); bronchodilator response ≥12% and ≥200 mL; treated with medium/high ICS + LABA; ≥2 exacerbations needing systemic corticosteroids in the previous year; ACQ-6 ≥1.5; weighing ≥40 kg | Exacerbation rate (primary analysis in patients with blood eosinophils ≥300 cells/µL and high dose ICS + LABA) | Q4W: rate ratio 0·55 (0·42–0·71) Q8W: 0·49  (0·37–0·64) | p<0·0001 |
| FitzGerald et al  CALIMA  2016 [14]  Phase 3  Lancet | 30 mg  Every 4 weeks or every 8 weeks  56 weeks  n=1306 | Adults and children (12-75 years old); pre-bronchodilator FEV1 ≤80% (adults) or ≤90% (children); bronchodilator response ≥12% and ≥200 mL; treated with fluticasone diproprionate >250 µg/day + LABA; ≥2 exacerbations needing systemic corticosteroids in the previous year; ACQ-6 ≥1.5; weighing ≥40 kg | Exacerbation rate (primary analysis in patients with blood eosinophils ≥300 cells/µL and high dose ICS + LABA) | Q4W: rate ratio :  0·64 (0·49-0·85)  Q8W: rate ratio:  0·72 (0·54-0·95) | p=0·0018  p=0·0188 |
| Park et al  2016 [15]  Phase 2  Int Arch Allergy Immunol | 2 mg, 20 mg or 100 mg  Every 8 weeks  52 weeks  n=106 | Adults (20-75 years old); pre-bronchodilator 40-90%; bronchodilator response ≥12% and ≥200 mL or PC_20_ ≤8 mg/mL; treated with medium/high ICS + LABA; 2-6 exacerbations needing systemic corticosteroids in the previous year; sputum eosinophils ≥2% or FeNO ≥50 ppb; ACQ-6 ≥1.5 | Exacerbation rate (percentage reduction) | 2mg: 33%  20mg: 45%  100ng:36% | No statistical  significance reported |
| Nowak et al  2015 [29]  Phase 2  Am J Emerg Med | 0.3 mg/kg or 1 mg/kg  Once during exacerbation  n=110 | Adults (18-60 years old) with an emerging asthma exacerbation lasting ≥2h treated with SABA and FEV_1_ ≤70%; ≥1 exacerbation in the previous year | Exacerbation rate 12 weeks after 1 IV benralizumab infusion during exacerbation(percent reduction) | 49% (3.59 vs 1.82) | P = .01 |
| Castro et al, 2014[30] Lancet Respir Med | Eosinophilic patients :  placebo, 2 mg benralizumab, 20 mg benralizumab, or 100 mg benralizumab Non-eosiophilic: | Adults aged 18-75 years with uncontrolled asthma using medium-dose or high-dose inhaled corticosteroids and long-acting β agonists, with two to six exacerbations in the past year. | Annual exacerbation rate in eosinophilic individuals after 1 year of follow-up | 100 mg group (0·34 vs 0·57, reduction 41%, 80% CI 11 to 60) (in eosinophilics) | p=0·096 |
| Ferguson et al., 2017[16] Lancet Respir Med | SC placebo or benralizumab 30 mg injections every 4 weeks for 12 weeks. | Patients aged 18-75 years, weighing at least 40 kg, and with a post-bronchodilator reversibility in forced expiratory volume in 1 s (FEV1) of at least 12% at screening, receiving either low- to medium-dosage inhaled corticosteroids (ICS) or low-dosage ICS plus long-acting β2 agonist fixed-combination therapy at screening, had a morning pre-bronchodilator FEV1 of more than 50% to 90% predicted at screening, and had one or more of the following symptoms within the 7 days before randomization: a daytime or night-time asthma symptom score of at least 1 for at least 2 days, rescue short-acting β2 agonist use for at least 2 days, or night-time awakenings due to asthma for at least one night. | Change from baseline pre-bronchodilator FEV1 at week 12. | 80mL (0-150) | p=0·04 |

*IL = interleukin, IV = intravenous, SC = subcutaneous, ICS = inhaled corticosteroids, FEV_1_ = forced expiratory volume in one second, FVC = forced vital capacity, FeNO = fractional exhaled nitric oxide, PEF = peak expiratory flow, PC_20_ = provocation concentration causing a 20% fall in FEV_1_, ACQ = Asthma Control Questionnaire, IL-5Rα = interleukin-5 receptor α, LABA = long-acting beta_2_-agonists, SABA = short-acting beta_2_- agonist.*

| Table 2: Targeting interleukin-13 in the treatment of asthma. | | | |  |  |
| --- | --- | --- | --- | --- | --- |
| TARGETING INTERLEUKIN-13 | | | |  |  |
| LEBRIKIZUMAB (anti-IL-13 / SC) | | | |  |  |
|  | **Intervention** | **Inclusion criteria** | **Primary outcomes** | **Effect (95%CI)** | **P value** |
| Hanania et al  LAVOLTA I, LAVOLTA II  2016 [19]  Phase 3  Lancet | 37.5 mg or 125 mg  Every 4 weeks  52 weeks  n = 2148 | Adults; pre-bronchodilator FEV_1_ 40%-80%; treaded with ICS plus ≥1 additional controller medication; uncontrolled asthma | Exacerbation rate (primary analysis in biomarker high patients: periostin ≥50 ng/mL or blood eosinophils ≥300 /µL) | LAVOLTA I:  37.5 mg: rate ratio  0·50 (0·37–0·67)  125mg: 0·70 (0·54–0·91) | <0·0001  0·0078 |
|  |  |  |  | LAVOLTA II  37.5 mg: rate ratio  0·86 (0·66–1·12)  125mg: 0·79 (0·61–1·04) | 0·2607  0·0920 |
| Hanania et al  LUTE and VERSE  2015 [17]  Phase 2  Thorax | 37.5 mg, 125 mg or 250 mg  Every 4 weeks  24 weeks  n=463 | Adults (18-75 years old); pre-bronchodilator FEV_1_ 40-80%; bronchodilator response ≥12%; treated with fluticasone propionate 500‑2000 µg/day or equivalent plus an additional controller medication; ACQ-5 score ≥1.5; one or more of the following: symptoms >2 days, ≥1 night awakenings, ≥2 reliever medications per week or interference with normal daily activities. | Exacerbation rate (percent reduction) | Periostin high : (all doses: 60% (18-80))  periostin-low patients (all doses: 5% reduction) |  |
| Noonan et al 2013 [20]  Phase 2  J Allergy Clin Immunol | 125 mg, 250 mg or 500 mg  Every 4 weeks  12 weeks  n=212 | Adults (18-65 years old); pre-bronchodilator FEV_1_ 60-65%; bronchodilator response ≥15%; with stable PEF; not treated with ICS or systemic glucocorticoids; SABA inhalations <10/day. | Change in FEV_1_ at week 12 | 125-mg dose group, 3.5% (−1.1% to 8.1%)  250-mg dose group: 4.8%( −0.1% to 9.7%)  500-mg dose group:  2.3% (−2.6% to 7.3%) | P = .13  P = .05  P = .35 |
| Corren et al 2011 [18]  Phase 2  N Engl J Med | 250 mg  Every 4 weeks  6 months  n=219 | Adults; pre-bronchodilator FEV_1_ 40-80%; bronchodilator response ≥12%; treated with fluticasone propionate 200-1000 µg/day or equivalent; ACQ-5 score ≥1.5 | Change in FEV_1_ at week 12 | 5.5% (0,8-10.2) | P=0.02 |
| TRALOKINUMAB (anti-IL-13 / SC) | | | |  |  |
|  | **Intervention** | **Inclusion criteria** | **Primary outcomes** | **Effect (95%CI)** | **P value** |
| Panettieri et al  STRATOS 1, STRATOS 2  2018 [31]  Phase 3  Lancet | 300 mg  Every 2 weeks or every 4 weeks  52 weeks  n = 2051 | Adults and children (12-75 years old); treated with fluticasone ≥ 500 µg/day or equivalent and LABA; uncontrolled asthma | Exacerbation rate | rate ratio 0∙93 [0∙72 to 1∙21] | p=0·59 |
| Piper et al 2013 [25]  Phase 2  Eur Respir J | 150 mg, 300 mg or 600 mg  Every 2 weeks  13 weeks  n=194 | Adults (18-65 years old); pre-bronchodilator FEV_1_ ≥40%; bronchodilator response ≥12% and ≥200 ml with BMI 18-40kg/m^2^; ACQ-6 score ≥1.5; had ≥1 exacerbation with medical intervention in the previous year | Change in ACQ-6 at week 13 | -0.76±1.04 | p=0.375 |
| Brightling et al 2015 [32]  Phase 2 b  Lancet Respir Med | 300mg every 2 weeks, 4 weeks or Placebo | Patients aged 18–75 years with severe asthma and two to six exacerbations in the previous year | annual asthma exacerbation rate at week 52(percent reduction) | 6% (–31 to 33) | p=0·709 |
| Russel et al, 2018[33] Lancet Respir Med | tralokinumab (300 mg) or placebo n=79 | 18-75 years with inadequately controlled moderate-to-severe asthma for 12 months or more, requiring treatment with inhaled corticosteroids at a stable dose. | change from baseline to week 12 in bronchial biopsy eosinophil count. | treatment effect ratio  1·43(0·63-3·27) | p=0·39 |
| GSK679586 (anti-IL-13 / IV) | | | |  |  |
|  | **Intervention** | **Inclusion criteria** | **Primary outcomes** |  |  |
| De Boever et al  2014 [26]  Phase 2  J Allergy Clin Immunol | 10mg/kg  Every 4 weeks  12 weeks  n=237 | Adults (18-75 years old); prebronchodilator FEV_1_ 35-80%; bronchodilator response ≥12%; treated with fluticasone propionate 1000 µg/day or equivalent; ACQ-7 score ≥1.5 | Change in ACQ-7 at week 12 | -0.31 teatment vs  placebo = -0.17 | P = 0.058 |

*IL = interleukin, SC = subcutaneous, FEV_1_ = forced expiratory volume in one second, ICS = inhaled corticosteroids, ACQ = Asthma Control Questionnaire, PEF = peak expiratory flow, SABA = short-acting beta_2_-agonist, LABA = long-acting beta_2_-agonist, BMI = body mass index, IV = intravenous.*

| Table 3: Targeting both interleukin-4 and interleukin-13 in the treatment of asthma. | | | |  |  |
| --- | --- | --- | --- | --- | --- |
| TARGETING BOTH INTERLEUKIN-4 AND INTERLEUKIN-13 | | | |  |  |
| DUPILUMAB (anti-IL-4Rα / SC) | | | |  |  |
|  | **Intervention** | **Inclusion criteria** | **Primary outcomes** | **Effect (95%CI)** | **P value** |
| Castro et al 2018 [21]  QUEST,  Phase 3  N Engl J Med | 200 mg or 300 mg  Every 2 weeks  52 weeks  n=1902 | Adults and children (≥12 years); pre-bronchodilator FEV_1_ ≤80% (>17 years) or ≤90% (12-17 years); bronchodilator response ≥12% and 200 ml; treated with fluticasone propionate ≥ 500 µg/day or equivalent and ≤2 additional controllers; systemic glucocorticoid treatment or hospitalization or emergency visit in the previous year; ACQ‑5 score ≥1.5 | Exacerbation rate;  Absolute FEV_1_ at week 12(percent reduction) | 200mg:  47.7% reduction in exacerbations rate  300 mg:  46.0% reduction in exacerbations rate | P<0.001 |
| Wenzel et al 2016 [23]  Phase 2 Lancet | 200 mg or 300 mg  Every 2 or 4 weeks  24 weeks  n=769 | Adults (≥18 years); pr-e-bronchodilator FEV_1_ 40-80%; bronchodilator response ≥12% and 200 ml; treated with fluticasone propionate ≥500 µg/day or equivalent plus LABA; had ≥1 systemic glucocorticoid treatment or hospitalization or emergency visit in the previous year; ACQ-5 score ≥1.5 | Absolute FEV_1_ at week 12 when eosinophils ≥300/µL | 300 mg :  mean difference (0·21 (0·06–0·36)  200 mg group:  mean difference 0·26 (0·11–0·40) | p=0·0063  p=0·0008 |
| Wenzel et al 2013 [22]  Phase 2;  N Engl J Med | 300 mg  Every week  12 weeks  n=104 | Adults (18-65 years); pre-bronchodilator FEV_1_ ≥50%; treated with fluticasone 500µg/day and salmeterol 100µg/day or equivalent; ); blood eosinophil count ≥300/µl or sputum eosinophils ≥3%; ACQ-5 score 1.5-3; had ≥1 systemic glucocorticoid treatment or hospitalization or emergency visit in the previous 2 years | Exacerbation rate | OR:  0.08 0.02 to 0.28 | P<0.001 |

*IL-4Rα = interleukin-4 receptor α, SC = subcutaneous, FEV_1_ = forced expiratory volume in one second, ACQ = Asthma Control Questionnaire, IV = intravenous.*

| Table 4: Targeting interleukin-9 in the treatment of asthma | | | |  |  |
| --- | --- | --- | --- | --- | --- |
| TARGETING INTERLEUKIN-9 | | | |  |  |
| MEDI-528 (anti-IL-9 / SC) | | | |  |  |
|  | **Intervention** | **Inclusion criteria** | **Primary outcomes** | **Effect (95%CI)** | **P value** |
| Oh et al  2013 [27]  Phase 2  Respir Res | 30 mg, 100 mg or 300 mg  Every 2 weeks  24 weeks  n=329 | Adults (18-65 years); pre-bronchodilator FEV_1_ ≥40%; bronchodilator response ≥12% and ≥200 mL; treated with medium/high dose ICS; ACQ-6 ≥1.5; daytime symptoms ≥2/week; night awakening ≥1/week; rescue medication use ≥2/week; ≥1 exacerbation in the previous year; BMI 18-35 kg/m^2^ | Change in ACQ-6 at week 13 | 2.8 (SD: 0.7)  2.8 (0.8) |  |

*IL = interleukin, SC = subcutaneous, FEV_1_ = forced expiratory volume in one second, ICS = inhaled corticosteroids, BMI = body mass index*

| Table 5: Targeting thymic stromal lymphopoietin in the treatment of asthma | | | |  |  |
| --- | --- | --- | --- | --- | --- |
| TARGETING THYMIC STROMAL LYMPHOPOIETIN | | | |  |  |
| TEZEPELUMAB (anti-TSLP / SC) | | | |  |  |
|  | **Intervention** | **Inclusion criteria** | **Primary outcomes** | **Effect (95%CI)** | **P value** |
| Corren et al  PATHWAY  2017 [24]  Phase 2  N Engl J Med | 70 mg, 210 mg or 280 mg  Every 4 weeks or every 2 weeks  52 weeks  n=584 | Adults (18-75 years); pre-bronchodilator FEV_1_ 40-80%; bronchodilator response ≥12% and ≥200 mL; treated with fluticasone ≥ 250 µg/day or equivalent; ≥ 2 exacerbations requiring systemic glucocorticoids or ≥ 1 exacerbation requiring hospitalization in the previous year; ACQ-6 ≥ 1.5 | Exacerbation rate(percent change vs placebo) | 70mg : 62%  210 mg : 71%  280 mg: 66% | P<0.001 |

*TSLP = thymic stromal lymphopoietin, SC = subcutaneous, FEV_1_* = forced expiratory volume in one second, ACQ = Asthma Control Questionnaire.

| Table 6 : Targeting CD25 | | | |  |  |
| --- | --- | --- | --- | --- | --- |
| Daclizumab | | | |  |  |
|  | **Intervention** | **Inclusion Criteria** | **Primary outcomes** | **Effect (95%CI)** | **P value** |
| Busse et al,  2008[34] | Daclizumab (intravenous loading dose, 2 mg/kg, then 1 mg/kg) | The primary inclusion criteria were: nonsmoking adults with asthma; 18–70 years old; asthma history of 6 months or longer; FEV1 of 50–80% of predicted; reversibility of at least 12% with inhaled short-acting β2-agonist; at least 1,200 μg daily inhaled TAA (or equivalent ICS) for 3 months or more before enrollment. | change in pulmonary function (percent change in FEV1) from randomization to Day 84. | 4.4 +/- 1.80% vs. placebo, 1.5 +/- 2.39% | P = 0.05 |

**Supplementary Figure 1:** Exacerbation rates among the different treatment arms, ordered by number of subjects treated

**
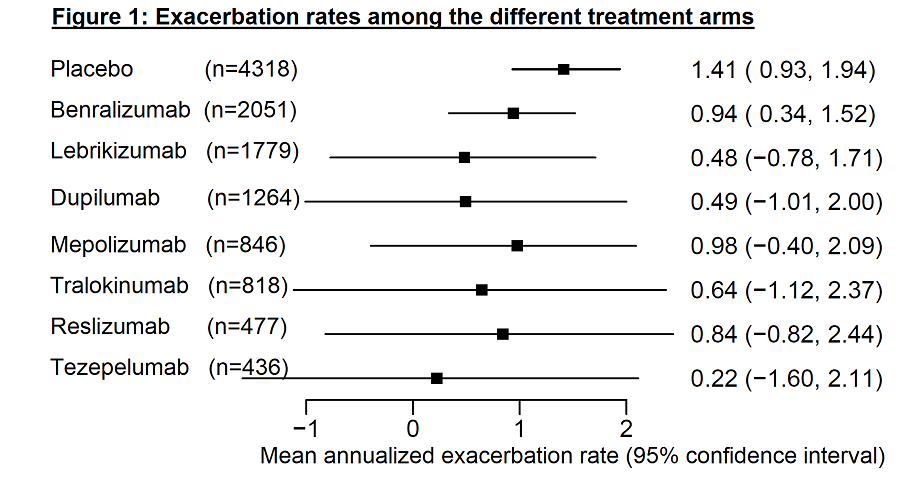
**

Median Annualized Exacerbation rate (95% CI)

Legend: Forest plot showing the exacerbation rates achieved with all included agents, very wide confidence intervals can be seen with all agents, and no statistically significant effects against placebo could be detected in the overall analysis.

**References**

1. Lin L, Zhang J, Hodges JS, Chu H. Performing Arm-Based Network Meta-Analysis in R with the pcnetmeta Package. J Stat Softw. 2017;80.

2. Team RC. R: A language and environment for statistical computing. Vienna, Austria: R Foundation for Statistical Computing,; 2018 [

3. Lajeunesse MJ. Facilitating systematic reviews, data extraction and meta-analysis with the metagear package for r. Methods Ecol Evol. 2016;7(3):323-30.

4. Ortega HG, Liu MC, Pavord ID, Brusselle GG, FitzGerald JM, Chetta A, et al. Mepolizumab treatment in patients with severe eosinophilic asthma. The New England journal of medicine. 2014;371(13):1198-207.

5. Pavord ID, Korn S, Howarth P, Bleecker ER, Buhl R, Keene ON, et al. Mepolizumab for severe eosinophilic asthma (DREAM): a multicentre, double-blind, placebo-controlled trial. The Lancet. 2012;380(9842):651-9.

6. Haldar P, Brightling CE, Hargadon B, Gupta S, Monteiro W, Sousa A, et al. Mepolizumab and exacerbations of refractory eosinophilic asthma. New England Journal of Medicine. 2009;360(10):973-84.

7. Chupp GL, Bradford ES, Albers FC, Bratton DJ, Wang-Jairaj J, Nelsen LM, et al. Efficacy of mepolizumab add-on therapy on health-related quality of life and markers of asthma control in severe eosinophilic asthma (MUSCA): a randomised, double-blind, placebo-controlled, parallel-group, multicentre, phase 3b trial. Lancet Respir Med. 2017;5(5):390-400.

8. Bjermer L, Lemiere C, Maspero J, Weiss S, Zangrilli J, Germinaro M. Reslizumab for Inadequately Controlled Asthma With Elevated Blood Eosinophil Levels: A Randomized Phase 3 Study. Chest. 2016;150(4):789-98.

9. Castro M, Zangrilli J, Wechsler ME, Bateman ED, Brusselle GG, Bardin P, et al. Reslizumab for inadequately controlled asthma with elevated blood eosinophil counts: results from two multicentre, parallel, double-blind, randomised, placebo-controlled, phase 3 trials. The Lancet Respiratory Medicine. 2015;3(5):355-66.

10. Castro M, Mathur S, Hargreave F, Boulet LP, Xie F, Young J, et al. Reslizumab for poorly controlled, eosinophilic asthma: a randomized, placebo-controlled study. American journal of respiratory and critical care medicine. 2011;184(10):1125-32.

11. Corren J, Weinstein S, Janka L, Zangrilli J, Garin M. Phase 3 Study of Reslizumab in Patients With Poorly Controlled Asthma: Effects Across a Broad Range of Eosinophil Counts. Chest. 2016;150(4):799-810.

12. Murphy K, Jacobs J, Bjermer L, Fahrenholz JM, Shalit Y, Garin M, et al. Long-term Safety and Efficacy of Reslizumab in Patients with Eosinophilic Asthma. J Aller Cl Imm-Pract. 2017;5(6):1572-81.

13. Bleecker ER, FitzGerald JM, Chanez P, Papi A, Weinstein SF, Barker P, et al. Efficacy and safety of benralizumab for patients with severe asthma uncontrolled with high-dosage inhaled corticosteroids and long-acting beta 2-agonists (SIROCCO): a randomised, multicentre, placebo-controlled phase 3 trial. Lancet. 2016;388(10056):2115-27.

14. FitzGerald JM, Bleecker ER, Nair P, Korn S, Ohta K, Lommatzsch M, et al. Benralizumab, an anti-interleukin-5 receptor a monoclonal antibody, as add-on treatment for patients with severe, uncontrolled, eosinophilic asthma (CALIMA): a randomised, double-blind, placebo-controlled phase 3 trial. Lancet. 2016;388(10056):2128-41.

15. Park HS, Kim MK, Imai N, Nakanishi T, Adachi M, Ohta K, et al. A Phase 2a Study of Benralizumab for Patients with Eosinophilic Asthma in South Korea and Japan. International archives of allergy and immunology. 2016;169(3):135-45.

16. Ferguson GT, FitzGerald JM, Bleecker ER, Laviolette M, Bernstein D, LaForce C, et al. Benralizumab for patients with mild to moderate, persistent asthma (BISE): a randomised, double-blind, placebo-controlled, phase 3 trial. Lancet Respir Med. 2017;5(7):568-76.

17. Hanania NA, Noonan M, Corren J, Korenblat P, Zheng Y, Fischer SK, et al. Lebrikizumab in moderate-to-severe asthma: pooled data from two randomised placebo-controlled studies. Thorax. 2015;70(8):748-56.

18. Corren J, Lemanske Jr RF, Hanania NA, Korenblat PE, Parsey MV, Arron JR, et al. Lebrikizumab treatment in adults with asthma. New England Journal of Medicine. 2011;365(12):1088-98.

19. Hanania NA, Korenblat P, Chapman KR, Bateman ED, Kopecky P, Paggiaro P, et al. Efficacy and safety of lebrikizumab in patients with uncontrolled asthma (LAVOLTA I and LAVOLTA II): replicate, phase 3, randomised, double-blind, placebo-controlled trials. The Lancet Respiratory Medicine. 2016;4(10):781-96.

20. Noonan M, Korenblat P, Mosesova S, Scheerens H, Arron JR, Zheng Y, et al. Dose-ranging study of lebrikizumab in asthmatic patients not receiving inhaled steroids. The Journal of allergy and clinical immunology. 2013;132(3):567-74.

21. Castro M, Corren J, Pavord ID, Maspero J, Wenzel S, Rabe KF, et al. Dupilumab Efficacy and Safety in Moderate-to-Severe Uncontrolled Asthma. The New England journal of medicine. 2018;378(26):2486-96.

22. Wenzel S, Ford L, Pearlman D, Spector S, Sher L, Skobieranda F, et al. Dupilumab in persistent asthma with elevated eosinophil levels. New England Journal of Medicine. 2013;368(26):2455-66.

23. Wenzel S, Castro M, Corren J, Maspero J, Wang L, Zhang BZ, et al. Dupilumab efficacy and safety in adults with uncontrolled persistent asthma despite use of medium-to-high-dose inhaled corticosteroids plus a long-acting beta(2) agonist: a randomised double-blind placebo-controlled pivotal phase 2b dose-ranging trial. Lancet. 2016;388(10039):31-44.

24. Corren J, Parnes JR, Wang L, Mo M, Roseti SL, Griffiths JM, et al. Tezepelumab in adults with uncontrolled asthma. New England Journal of Medicine. 2017;377(10):936-46.

25. Piper E, Brightling C, Niven R, Oh C, Faggioni R, Poon K, et al. A phase II placebo-controlled study of tralokinumab in moderate-to-severe asthma. The European respiratory journal. 2013;41(2):330-8.

26. De Boever EH, Ashman C, Cahn AP, Locantore NW, Overend P, Pouliquen IJ, et al. Efficacy and safety of an anti-IL-13 mAb in patients with severe asthma: A randomized trial. The Journal of Allergy and Clinical Immunology. 2014;133(4):989-96.

27. Oh CK, Leigh R, McLaurin KK, Kim K, Hultquist M, Molfino NA. A randomized, controlled trial to evaluate the effect of an anti-interleukin-9 monoclonal antibody in adults with uncontrolled asthma2013 17 August 2018; 14. Available from: <https://doi.org/10.1186/1465-9921-14-93>.

28. Flood-Page P, Swenson C, Faiferman I, Matthews J, Williams M, Brannick L, et al. A study to evaluate safety and efficacy of mepolizumab in patients with moderate persistent asthma. American journal of respiratory and critical care medicine. 2007;176(11):1062-71.

29. Nowak RM, Parker JM, Silverman RA, Rowe BH, Smithline H, Khan F, et al. A randomized trial of benralizumab, an antiinterleukin 5 receptor alpha monoclonal antibody, after acute asthma. The American journal of emergency medicine. 2015;33(1):14-20.

30. Castro M, Wenzel SE, Bleecker ER, Pizzichini E, Kuna P, Busse WW, et al. Benralizumab, an anti-interleukin 5 receptor alpha monoclonal antibody, versus placebo for uncontrolled eosinophilic asthma: a phase 2b randomised dose-ranging study. Lancet Respir Med. 2014;2(11):879-90.

31. Panettieri Jr RA, Sjöbring U, Péterffy A, Wessman P, Bowen K, Piper E, et al. Tralokinumab for severe, uncontrolled asthma (STRATOS 1 and STRATOS 2): two randomised, double-blind, placebo-controlled, phase 3 clinical trials. The Lancet Respiratory Medicine. 2018;6(7):511-25.

32. Brightling CE, Chanez P, Leigh R, O'Byrne PM, Korn S, She D, et al. Efficacy and safety of tralokinumab in patients with severe uncontrolled asthma: a randomised, double-blind, placebo-controlled, phase 2b trial. Lancet Respir Med. 2015;3(9):692-701.

33. Russell RJ, Chachi L, FitzGerald JM, Backer V, Olivenstein R, Titlestad IL, et al. Effect of tralokinumab, an interleukin-13 neutralising monoclonal antibody, on eosinophilic airway inflammation in uncontrolled moderate-to-severe asthma (MESOS): a multicentre, double-blind, randomised, placebo-controlled phase 2 trial. Lancet Respir Med. 2018;6(7):499-510.

34. Busse WW, Israel E, Nelson HS, Baker JW, Charous BL, Young DY, et al. Daclizumab improves asthma control in patients with moderate to severe persistent asthma: a randomized, controlled trial. Am J Respir Crit Care Med. 2008;178(10):1002-8.
